# Targeted Therapy in Pancreatic Ductal Adenocarcinoma: Current Advances and Challenges

**DOI:** 10.3390/curroncol33080452

**Published:** 2026-07-28

**Authors:** Ramy Habib, Erika Arnold, Tasin Obi, Franco J. Vizeacoumar, Shahid Ahmed

**Affiliations:** 1 School of Medicine, University of Limerick, Limerick V94 T9PX, Munster, Ireland; 23316373@studentmail.ul.ie (R.H.); erika.arnold@mail.mcgill.ca (E.A.); 2 Department of Oncology, College of Medicine, University of Saskatchewan, Saskatoon, SK S7N 5E5, Canada; bnv137@mail.usask.ca (T.O.); franco.vizeacoumar@usask.ca (F.J.V.); 3 Saskatchewan Cancer Agency, Regina, SK S4W 0G3, Canada

**Keywords:** pancreatic ductal adenocarcinoma, PDAC, targeted therapy, precision oncology, KRAS, KRAS inhibitors, molecular profiling, PARP inhibitors, immunotherapy, liquid biopsy

## Abstract

**Background**: Pancreatic ductal adenocarcinoma (PDAC) remains one of the most lethal solid malignancies, with poor survival driven by late presentation, aggressive tumor biology, and limited responsiveness to conventional systemic therapy. Advances in molecular profiling have expanded opportunities for biomarker-guided and targeted therapeutic approaches. **Methods**: A literature review was conducted using PubMed and the Cochrane Library through July 2026, supplemented by abstracts and proceedings from major international oncology conferences. **Results**: Pancreatic cancer is driven mainly by somatic changes in KRAS, TP53, CDKN2A, and SMAD4. Established precision approaches include maintenance olaparib for selected platinum-sensitive tumors with germline BRCA1 or BRCA2 pathogenic variants, immune checkpoint inhibition for mismatch repair-deficient or microsatellite instability-high tumors, and tropomyosin receptor kinase inhibition for cancers with neurotrophic tyrosine receptor kinase gene fusions. Direct inhibition of KRAS and RAS represents a major therapeutic breakthrough. KRAS G12C inhibitors established proof of concept, while agents targeting the more common KRAS G12D mutation are showing encouraging early activity. In the randomized phase III RASolute 302 trial, the multiselective RAS inhibitor daraxonrasib improved survival compared with chemotherapy in previously treated metastatic disease with oncogenic RAS mutations. Early studies of zoldonrasib combinations have extended this progress to KRAS G12D-mutant disease, although confirmation is required. Molecular profiling, next-generation sequencing, patient-derived organoids, and circulating tumor DNA may further improve treatment selection and monitoring. **Conclusions**: Precision oncology is becoming clinically relevant in pancreatic ductal adenocarcinoma. KRAS- and RAS-directed therapies are central advances, but resistance, toxicity, limited durability, and access to comprehensive testing remain important challenges.

## Simple Summary

Pancreatic ductal adenocarcinoma (PDAC) is one of the deadliest forms of cancer and remains difficult to treat because it is often diagnosed at an advanced stage and responds poorly to standard treatments. Recent advances in genetic and molecular testing have improved our understanding of the biological changes that drive pancreatic cancer and have created new opportunities for more personalized treatment approaches. This review summarizes current and emerging targeted therapies for PDAC. Established treatments include PARP inhibitors for patients with BRCA1, BRCA2, or PALB2 pathogenic variants, immunotherapy for rare tumors with mismatch repair deficiency, and TRK inhibitors for cancers with NTRK gene fusions. New therapies that directly target KRAS and RAS, key cancer-driving proteins found in most pancreatic cancers, represent a major breakthrough and are showing promising results. A randomized phase III trial demonstrated that daraxonrasib improved survival compared with chemotherapy in previously treated RAS-mutant metastatic PDAC, providing strong clinical evidence that RAS can be successfully targeted. Other KRAS-directed agents, including therapies for the common KRAS G12D mutation, are also advancing in clinical trials. Continued advances in molecular testing and targeted therapies may help expand treatment options and improve outcomes for patients with pancreatic cancer.

## 1. Introduction

Pancreatic ductal adenocarcinoma (PDAC) remains one of the most lethal solid malignancies and continues to impose a disproportionate mortality burden relative to its incidence. Globally, pancreatic cancer accounted for an estimated 510,992 new cases and 467,409 deaths in 2022, ranking twelfth in incidence but sixth for cancer-related mortality worldwide [1,2]. In the United States, an estimated 67,530 new cases and 52,740 deaths are projected in 2026, and pancreatic cancer is expected to become the second leading cause of cancer-related death by 2030 [3,4].

Prognosis remains poor largely because most patients present with advanced disease, precluding curative-intent resection. Approximately 80% of patients are diagnosed with either locally advanced unresectable or metastatic disease at presentation [5]. The overall 5-year relative survival is approximately 13.3%, declining to 3.2% for distant-stage disease, with over half of cases diagnosed after metastatic spread [5]. Even among patients who undergo surgical resection, recurrence remains common [6].

Systemic treatment of advanced PDAC has historically relied on cytotoxic chemotherapy. Current standard approaches include adjuvant modified FOLFIRINOX for patients with resected disease and good performance status and multi-agent regimens such as FOLFIRINOX, gemcitabine plus nab-paclitaxel, or NALIRIFOX in advanced disease [7,8,9,10,11]. Although these regimens have improved survival outcomes, median overall survival in metastatic PDAC generally remains less than one year despite modern combination chemotherapy [9,12,13,14,15].

Therapeutic efficacy is further limited by the unique biology of PDAC, including a dense hypovascular desmoplastic stroma, impaired drug delivery, immune suppression, and substantial intratumoral heterogeneity [16,17,18,19]. In addition, cytotoxic chemotherapy is associated with significant toxicities, including myelosuppression, mucositis, and neuropathy, which frequently compromise treatment tolerability [9,14,20].

These limitations have driven increasing interest in biomarker-guided and molecularly targeted therapies. This narrative review summarizes the molecular landscape of PDAC, current targeted therapeutic strategies, biomarker testing approaches, resistance mechanisms, and future directions in precision oncology for pancreatic cancer.

## 2. Methods

A literature review was conducted using structured searches of PubMed and the Cochrane Library from database inception to July 2026. Search terms included “pancreatic adenocarcinoma,” “pancreatic cancer,” “targeted therapy,” “precision oncology,” “KRAS inhibitors,” “PARP inhibitors,” and “clinical trials.” In addition to full-text publications, relevant conference proceedings and abstracts from major oncology meetings, including American Society of Clinical Oncology (ASCO), European Society for Medical Oncology (ESMO), and American Association for Cancer Research (AACR) were reviewed to capture recent and emerging clinical trial data.

## 3. Molecular Landscape of Pancreatic Cancer

### 3.1. Genomic Terminology and Inherited Versus Somatic Changes

PDAC genomic findings include sequence variants (single-nucleotide variants and small insertions or deletions), copy-number changes (amplifications or deletions), structural rearrangements and gene fusions, and epigenetic events such as promoter methylation. In this review, “genomic alteration” is retained only as an umbrella term when several event classes are included; otherwise, the more specific terms—mutation, pathogenic variant, amplification, deletion, fusion, or methylation—are used. Germline pathogenic variants are inherited and present in constitutional DNA, whereas somatic variants are acquired in tumor cells. A single-nucleotide polymorphism (SNP) generally denotes a common single-base variant and is not interchangeable with a pathogenic germline variant. This distinction is clinically important because pathogenic variants in BRCA1/2, PALB2, CDKN2A, and mismatch-repair genes may be germline or somatic, whereas most KRAS, TP53, and SMAD4 driver mutations in PDAC are somatic [21,22,23,24,25,26,27,28,29].

### 3.2. Common Somatic Driver Events

Current sequencing studies consistently identify four genes recurrently affected by somatic driver events in PDAC: KRAS, TP53, CDKN2A, and SMAD4 [21,22]. The Cancer Genome Atlas reported KRAS mutations in 93% of tumors, TP53 mutations in 72%, CDKN2A mutations or deletions in approximately 30%, and SMAD4 mutations or deletions in approximately 32%, findings reproduced across multiple cohorts, including early-onset disease [21,30,31,32]. These percentages are not mutually exclusive: KRAS-mutant tumors frequently acquire concurrent TP53, CDKN2A, and SMAD4 inactivation during progression. Co-occurring KRAS and TP53 mutations are especially common and are associated with more aggressive biology and poorer outcomes.

KRAS is the dominant oncogenic driver in PDAC, most commonly involving codons 12, 13, and 61, including G12D, G12V, G12R, and less commonly G12C mutations [21,33,34,35]. These somatic mutations promote constitutive MAPK and PI3K signaling and are associated with aggressive tumor biology and worse survival [21,22,36,37]. Approximately 7–10% of PDAC is KRAS wild type. Alternative actionable events in this subgroup must be reported with their denominator: in unselected PDAC, ERBB2 amplification occurs in approximately 1–2%, BRAF mutations or fusions in approximately 2–3%, and NTRK fusions in less than 1%; FGFR2 fusions or rearrangements are generally uncommon overall but have been reported in approximately 5–7% of selected KRAS-wild-type cohorts [21,38,39,40,41]. This enrichment makes comprehensive DNA- and RNA-based profiling particularly important in KRAS-wild-type tumors.

TP53 is the most frequently mutated tumor suppressor gene in PDAC and contributes to impaired DNA-damage response, apoptosis resistance, chromosomal instability, and chemoresistance [21,22,33,42]. Clinically, TP53 mutations, especially when co-occurring with KRAS mutations, are associated with worse outcomes [43,44,45].

CDKN2A is inactivated through somatic mutation, homozygous deletion, or promoter hypermethylation in approximately 40–60% of PDAC when all mechanisms are considered, resulting in dysregulated cell-cycle control [21,30,46]. Rates closer to 30% generally reflect sequence mutation and deletion estimates in particular genomic datasets, whereas broader estimates include epigenetic silencing. Rare germline CDKN2A pathogenic variants associated with familial atypical multiple mole melanoma syndrome confer a markedly increased PDAC risk [47,48,49].

SMAD4 (DPC4) is inactivated by somatic mutation or deletion in approximately 32% of PDAC, disrupting TGF-β signaling and associating with metastatic progression and poor prognosis [30,31,42,43,46,50]. Collectively, these canonical somatic driver events define major aspects of PDAC biology and contribute to its aggressive clinical behavior.

### 3.3. Chronic Pancreatitis Susceptibility Genes and PDAC Risk

Chronic pancreatitis is an established risk state for PDAC, and hereditary pancreatitis caused by pathogenic PRSS1 variants confers a particularly high lifetime risk. Pathogenic variants in SPINK1, CFTR, CTRC, CPA1, and related genes predispose individuals to recurrent or chronic pancreatitis, but their direct associations with PDAC are less uniform and may be mediated largely through longstanding inflammation. A systematic review and meta-analysis found a modest increase in pancreatic cancer risk with CFTR variants but no clear pooled association for SPINK1. These pancreatitis-susceptibility variants should therefore not be described as direct oncogenic drivers; rather, they identify inherited predisposition to pancreatic inflammation and, in selected high-risk individuals, support genetic counseling and surveillance [51,52].

### 3.4. Actionable Genomic Events

Although PDAC is largely defined by canonical somatic drivers, comprehensive genomic profiling identifies potentially actionable findings in approximately 12–25% of tumors, although fewer patients ultimately receive matched therapy because of tissue, access, clinical-trial, and disease-progression constraints [53,54,55]. Actionable findings include sequence mutations, copy-number amplifications, gene fusions, and DNA-repair or microsatellite-instability phenotypes. They are enriched in KRAS-wild-type tumors but also occur in KRAS-mutant disease, particularly homologous-recombination repair deficiency and the uncommon KRAS G12C mutation [38,39,56].

KRAS G12C, present in approximately 1–2% of PDAC, is the first directly targetable KRAS sequence mutation in this disease [21,23,34,57,58]. More prevalent KRAS G12D and G12V mutations, along with broader pan-KRAS and pan-RAS strategies, remain active areas of investigation and are discussed further in Section 5.1.4 [59,60,61,62].

Pathogenic variants in BRCA1, BRCA2, and PALB2 may be germline or somatic and define the most established DNA-damage-repair subgroup in PDAC, accounting collectively for approximately 5–10% of cases [23,63,64]. These variants impair homologous recombination repair and increase sensitivity to platinum chemotherapy and PARP inhibition [65,66,67]. The phase III POLO trial established maintenance olaparib for platinum-sensitive metastatic PDAC with a germline BRCA1/2 pathogenic variant, while evidence for somatic BRCA1/2, PALB2, and broader HRD populations remains less definitive [56,63,64,68].

Somatic NTRK1/2/3 gene fusions occur in less than 1% of PDAC but represent highly actionable oncogenic drivers, particularly in KRAS-wild-type tumors [39,60,69,70]. TRK inhibitors such as larotrectinib and entrectinib have demonstrated high and durable response rates across tumor types, although PDAC-specific numbers remain very small [69,70]. Detection is optimized by comprehensive DNA- and RNA-based profiling [23,39,60].

MSI-H/dMMR is a genomic phenotype present in approximately 1–3% of PDAC and may arise through somatic mismatch-repair inactivation or, less commonly, an inherited mismatch-repair pathogenic variant [56,71,72]. Pembrolizumab has tumor-agnostic activity in this setting, although PDAC responses appear less frequent than in several other MSI-H malignancies [71,72,73]. The dense, immunosuppressive PDAC microenvironment may limit effective immune infiltration despite elevated neoantigen burden.

Collectively, these findings highlight the molecular heterogeneity of PDAC and the growing role of biomarker-guided therapy. Established approaches include PARP inhibition for selected BRCA-associated tumors, immune checkpoint inhibition for MSI-H/dMMR disease, and TRK inhibition for NTRK fusion-positive cancers; emerging KRAS- and RAS-directed therapies may expand this scope but require attention to toxicity and resistance [57,63,70,71]. Key event classes, frequencies, clinical relevance, and example agents are summarized in Table 1.

### 3.5. Implications for Therapeutic Targeting

The molecular landscape of PDAC has important therapeutic implications. Canonical somatic mutations and tumor-suppressor inactivation involving KRAS, TP53, CDKN2A, and SMAD4 contribute to aggressive biology and resistance, whereas selected pathogenic variants, amplifications, fusions, and genomic phenotypes support biomarker-guided therapy [21,22,62]. Consolidating the biology and clinical evidence by molecular subgroup improves clarity, but clinical benefit remains limited by intratumoral heterogeneity, drug delivery barriers, adaptive resistance, and the small size of many actionable subgroups [53,54,56].

## 4. Rationale for Targeted Therapy

Conventional cytotoxic chemotherapy exerts antitumor effects through non-specific inhibition of rapidly proliferating cells, often resulting in significant toxicity to normal tissues [78]. In contrast, targeted therapies and immunotherapy aim to inhibit specific molecular pathways and enhance antitumor immune responses through biomarker-guided approaches [79,80].

Increasing understanding of the genomic and molecular landscape of PDAC has demonstrated that the disease is molecularly heterogeneous and contains selected subgroups with potentially actionable therapeutic vulnerabilities [81,82]. Transcriptomic analyses have further identified two major biologically distinct PDAC subtypes: the classical/progenitor subtype, characterized by epithelial differentiation and relatively better prognosis, and the basal-like/squamous subtype, which demonstrates more aggressive behavior, chemoresistance, and poorer survival outcomes [81,83,84]. These transcriptional subgroups may also influence therapeutic responsiveness and are increasingly recognized as important determinants of precision oncology strategies in PDAC. Molecular profiling studies further support the clinical relevance of biomarker-guided therapy by demonstrating improved outcomes in selected patients receiving matched treatments, leading to increasing integration of germline (BRCA1, BRCA2, PALB2) and somatic (KRAS, TP53, NRG1 fusions, etc.) testing into treatment selection and clinical trial enrollment strategies [53,85,86,87].

Despite this rationale, the effectiveness of targeted therapy in PDAC remains limited by dense desmoplastic stroma, hypovascularity, intratumoral heterogeneity, immunosuppressive myeloid compartments, and adaptive resistance mechanisms that impair drug delivery and therapeutic response [88,89,90]. Prior and emerging attempts to remodel fibrosis and immune suppression are reviewed in Section 5.2.4.

## 5. Clinical Evidence and Therapeutic Strategies

### 5.1. Clinically Relevant Targeted Therapies

Clinically validated targeted therapies in pancreatic ductal adenocarcinoma (PDAC) remain limited to relatively small molecularly defined subgroups despite broader advances in precision oncology [22,87,91]. Established approaches include PARP inhibition for tumors with pathogenic BRCA1/2 variants, immune checkpoint blockade for MSI-H/dMMR disease, TRK inhibition for NTRK fusion-positive cancers, and the modest benefit of erlotinib plus gemcitabine [87,91,92]. Emerging KRAS-directed therapies may further expand the therapeutic landscape, although most remain investigational and resistance continues to limit durable responses [59]. Established and emerging targeted therapeutic strategies and key clinical trial outcomes in PDAC are summarized in Table 2.

#### 5.1.1. PARP Inhibitors in BRCA/PALB2 Pathogenic-Variant PDAC (POLO and Beyond)

Pathogenic variants in homologous recombination repair (HRR) genes, especially BRCA1, BRCA2, and PALB2, define one of the most clinically relevant molecular subgroups in PDAC, accounting for approximately 5–10% of cases [91,101,102,103,104]. These variants may be germline or somatic, impair homologous recombination repair, and increase sensitivity to platinum chemotherapy and PARP inhibition [63,104,105,106].

The phase III POLO trial evaluated maintenance olaparib in metastatic PDAC with a germline pathogenic BRCA1/2 variant following platinum-based chemotherapy and demonstrated significantly improved progression-free survival compared with placebo (7.4 vs. 3.8 months; HR 0.53, 95% CI 0.35–0.82), although no statistically significant overall survival benefit was observed [63,107]. Treatment was generally well tolerated, with fatigue, nausea, and anemia as the most common adverse events [107].

Based on these findings, maintenance olaparib is a standard-of-care option for patients with platinum-sensitive metastatic PDAC harboring a germline pathogenic BRCA1/2 variant [63,86,94]. Additional studies suggest activity of other PARP inhibitors, including rucaparib, in disease with germline or somatic pathogenic BRCA1/2 or PALB2 variants, while ongoing trials are evaluating combination strategies with immunotherapy and other targeted agents [64,87,91,95].

Overall, PARP inhibition remains the most established biomarker-guided targeted therapy in PDAC, although challenges related to resistance, optimal patient selection, and extension to broader HRD populations remain unresolved [56,91].

#### 5.1.2. EGFR Inhibition: Erlotinib

EGFR signaling was an early therapeutic target in PDAC, leading to evaluation of erlotinib in combination with gemcitabine [91,96]. In the phase III PA.3 trial, erlotinib plus gemcitabine demonstrated statistically significant improvements in overall survival (6.24 vs. 5.91 months; HR 0.82, 95% CI 0.69–0.99), one-year survival, and progression-free survival compared with gemcitabine alone, but with increased rash and diarrhea [96].

Despite statistical significance, the absolute survival benefit was minimal, and subsequent studies and meta-analyses failed to demonstrate meaningful additional clinical benefit while confirming increased toxicity [91,108,109]. Consequently, erlotinib–gemcitabine is no longer a preferred first-line regimen, with current treatment standards favoring multi-agent chemotherapy approaches such as modified FOLFIRINOX, gemcitabine–nab-paclitaxel, and NALIRIFOX [11,110]. Erlotinib is therefore best viewed as an early proof-of-concept for targeted therapy in PDAC rather than a clinically transformative strategy [56,91].

#### 5.1.3. NTRK Fusion-Directed Therapy: Larotrectinib and Entrectinib

NTRK1/2/3 fusions are rare in PDAC (<1%) but represent actionable oncogenic drivers, particularly in KRAS wild-type tumors [11,87]. TRK inhibitors such as larotrectinib and entrectinib have demonstrated high and durable response rates across NTRK fusion-positive solid tumors, leading to tumor-agnostic approvals despite limited PDAC-specific data [70,87,97].

Pooled analyses of larotrectinib studies (LOXO-TRK-14001, SCOUT, NAVIGATE) demonstrated objective response rates of approximately 75–80% across tumor types, including durable responses in the small number of PDAC patients included [87,97]. Case reports have also described rapid responses followed by acquired resistance through secondary kinase-domain mutations, with subsequent activity of next-generation TRK inhibitors [87,111]. Entrectinib has shown similar histology-agnostic activity, including partial responses and prolonged disease control in limited PDAC cases [87,98].

Accordingly, larotrectinib and entrectinib are recommended for unresectable or metastatic NTRK fusion-positive PDAC after standard chemotherapy when the fusion is confirmed by validated molecular testing [56,87,98,111,112]. Although only a small minority of patients benefit, these therapies illustrate the importance of comprehensive molecular profiling, especially in KRAS wild-type PDAC [56,87].

#### 5.1.4. RAS-Directed Therapy: Emerging KRAS and Pan-RAS Inhibitors

KRAS mutations occur in more than 90% of PDAC [113]. KRAS G12D is the most common subtype, accounting for approximately 42% of KRAS-mutant tumors, followed by KRAS G12V at approximately 32%, whereas KRAS G12C occurs in only 1–2% [59,74].

Clinical proof-of-concept for direct KRAS inhibition was established with KRAS G12C inhibitors such as sotorasib and adagrasib, which demonstrated objective responses and disease control across KRAS G12C-mutant solid tumors, including PDAC [57,58]. Although KRAS G12C mutations are uncommon in pancreatic cancer, these studies validated KRAS as a therapeutically targetable oncogenic driver and accelerated the development of broader KRAS-directed strategies.

Given the high prevalence of KRAS G12D mutations in PDAC, development is increasingly focused on allele-specific inhibitors and degraders. Zoldonrasib (RMC-9805) is an oral, covalent RAS(ON) G12D-selective tri-complex inhibitor, whereas MRTX1133 and ASP3082 represent additional small-molecule or degrader approaches in development [74,75,76,77]. Although these agents provide a rational strategy for a large molecular subgroup of PDAC, their clinical roles remain investigational.

Early-phase studies suggest activity of zoldonrasib combinations in RAS G12D-mutant metastatic PDAC. In RMC-GI-102, zoldonrasib plus modified FOLFIRINOX or gemcitabine/nab-paclitaxel achieved objective response rates of 82% and 61%, respectively, among efficacy-evaluable patients [76]. In RMC-9805-001, zoldonrasib plus daraxonrasib achieved response rates of 50% in the second-line cohort and 47% in later lines, with median progression-free survival of 9.6 and 7.6 months, respectively [77]. Grade ≥ 3 treatment-related adverse events occurred in 61–80% with chemotherapy and 35% with the targeted doublet. These small, single-arm studies remain hypothesis-generating. The randomized phase III RASolute 305 trial is evaluating zoldonrasib plus chemotherapy in previously untreated metastatic disease [100].

An alternative strategy involves pan-RAS inhibition, which aims to target multiple KRAS variants simultaneously. The RAS(ON) multi-selective inhibitor daraxonrasib (formerly RMC-6236) has demonstrated preliminary antitumor activity and disease control in KRAS-mutant PDAC in early-phase studies [114,115]. Subsequent clinical studies demonstrated encouraging activity both as monotherapy and in combination with gemcitabine plus nab-paclitaxel in RAS-mutant PDAC, supporting further evaluation in registrational trials [93,99,116].

The randomized phase III RASolute 302 trial compared daraxonrasib with investigator’s-choice chemotherapy in previously treated metastatic PDAC harboring oncogenic RAS mutations [93]. Daraxonrasib improved median overall survival (13.2 vs. 6.7 months; hazard ratio for death, 0.40) and also improved progression-free survival, objective response rate, and duration of response. Safety requires balanced interpretation: grade 3 or higher adverse events occurred in 61.8% of patients receiving daraxonrasib and 69.6% receiving chemotherapy; treatment-related grade 3 or higher events occurred in 43.6% and 57.5%, respectively. Treatment-related adverse events prompted dose reduction in 36.1% and 57.5% and discontinuation in 1.2% and 11.2%, respectively. Rash and stomatitis were prominent toxicities requiring proactive supportive care and dose modification [93].

These results provide important randomized validation that multiselective RAS inhibition can improve survival in previously treated RAS-mutant metastatic PDAC [93]. A median overall survival of 13.2 months represents a clinically meaningful outcome in this difficult-to-treat setting, although longer follow-up is needed to define the durability of benefit, mechanisms of acquired resistance, and late toxicity. Daraxonrasib remains investigational and, at the time of this revision, is available through expanded access rather than full regulatory approval. Its eventual role in routine care will depend on regulatory approval, guideline incorporation, and confirmation of safety and effectiveness in real-world practice.

Despite these advances, several challenges continue to limit the impact of RAS-directed therapy in PDAC. Responses may be constrained by secondary RAS events, pathway reactivation, parallel signaling, phenotypic plasticity, and intratumoral heterogeneity [113,117,118]. Consequently, ongoing studies are evaluating rational combinations that inhibit SHP2, SOS1, MAPK, or PI3K signaling and other resistance nodes; these strategies remain investigational and require careful toxicity assessment [117,118].

Overall, RAS-directed therapy represents a major breakthrough and a rapidly evolving treatment strategy in PDAC. The phase III daraxonrasib data provide landmark randomized evidence that multiselective RAS inhibition can achieve clinically meaningful survival benefit in previously treated metastatic disease, establishing robust clinical proof-of-concept that oncogenic RAS can be effectively targeted in pancreatic cancer. Although further improvements in durability, toxicity, biomarker selection, and efficacy in earlier treatment settings are needed, these findings mark an important advance in precision oncology and may substantially broaden the future role of allele-specific and multiselective RAS-directed therapies in PDAC.

### 5.2. Targeted Therapies with Limited or Negative Results

Multiple targeted agents evaluated in phase II–III PDAC trials have failed to improve overall survival when added to chemotherapy and were often limited by toxicity, adaptive resistance, and pathway redundancy [87,91,119,120]. Anti-VEGF therapies, MEK inhibitors, mTOR inhibitors, stromal-targeting agents, and epigenetic therapies have generally demonstrated limited or negative results and are not recommended outside clinical trials [11,91,120]. These findings shifted the field toward biomarker-driven and rational combination strategies designed to overcome stromal, genomic, and immune resistance. Selected targeted therapies with limited or negative clinical results in PDAC are summarized in Table 3.

#### 5.2.1. Anti-VEGF Agents

Despite frequent VEGF overexpression in PDAC, anti-angiogenic strategies have failed to produce meaningful clinical benefit [119]. In the phase III CALGB 80303 and AViTA trials, bevacizumab-based combinations did not improve overall survival despite modest progression-free survival improvements and increased toxicity [119,121,122]. Similarly, VEGFR tyrosine kinase inhibitors including axitinib, sorafenib, pazopanib, regorafenib, and nintedanib failed to improve outcomes in advanced PDAC [119]. These negative results likely reflect the predominantly desmoplastic, hypovascular, hypoxic, and immunosuppressive PDAC microenvironment, which is not adequately reversed by VEGF blockade [119].

#### 5.2.2. MEK Inhibition

Given the central role of KRAS-driven MAPK signaling in PDAC, MEK inhibition initially appeared promising. However, clinical studies of selumetinib and trametinib, including a randomized trial evaluating trametinib in combination with gemcitabine, failed to improve survival outcomes and were associated with increased toxicity [119,123,124,125]. Similarly, the combination of selumetinib and the AKT inhibitor MK-2206 in the SWOG S1115 trial demonstrated limited efficacy and greater toxicity compared with chemotherapy [125]. Resistance to MEK inhibition is likely driven by compensatory signaling pathways and the molecular heterogeneity of KRAS-driven disease [119,120].

#### 5.2.3. mTOR Inhibitors

Although the PI3K-AKT-mTOR pathway is frequently activated in PDAC, mTOR inhibitors such as everolimus have demonstrated minimal clinical activity in both monotherapy and combination studies, with additional metabolic and hematologic toxicity [120,126,127]. These negative results are thought to reflect feedback activation of parallel signaling pathways and stromal barriers to drug delivery [120].

#### 5.2.4. Extracellular Matrix and Stromal Targeting

Targeting the dense stromal and immunosuppressive microenvironment has produced mixed, predominantly negative clinical results. PEGPH20 initially showed activity signals in hyaluronan-high PDAC, but the phase III HALO-301 trial failed to improve overall survival, illustrating that broad extracellular-matrix depletion is insufficient and may disrupt restraining stromal functions [128,129]. The CCR2 inhibitor PF-04136309 reduced circulating inflammatory monocytes when combined with gemcitabine/nab-paclitaxel, but it produced no efficacy signal above chemotherapy and raised concern for synergistic pulmonary toxicity [130]. In the phase III SEQUOIA trial, the pegylated interleukin-10 agonist pegilodecakin added to FOLFOX did not improve overall or progression-free survival and increased hematologic and constitutional toxicity [131]. More recently, the six-patient phase I PROGEM study found that proglumide could be combined with gemcitabine/nab-paclitaxel without a clear drug-related safety signal and reported exploratory blood-biomarker changes consistent with reduced fibrosis and metastasis; however, its nonrandomized sample is far too small to establish antitumor efficacy [132]. Together, these studies support selective, biomarker-informed remodeling of stromal and immune compartments rather than nonspecific stromal depletion.

#### 5.2.5. Epigenetic Therapies: HDAC Inhibitors

HDAC inhibitors demonstrated promising preclinical activity in PDAC; however, clinical studies showed limited efficacy and significant toxicity, preventing clinical adoption outside trials [133].

### 5.3. Combination Strategies

Combination approaches aim to improve the limited efficacy of single-agent targeted or immune therapies in PDAC by enhancing chemotherapy activity and modulating the tumor microenvironment [88,134,135]. These strategies target key features of PDAC biology, including stromal barriers, immune suppression, impaired drug delivery, and adaptive resistance. Although most data remain early phase and no combination has yet established a new standard of care, combination strategies remain a major focus of ongoing investigation.

#### 5.3.1. Targeted Plus Chemotherapy

Targeted therapy combined with chemotherapy is being investigated to enhance tumor vulnerability while suppressing compensatory survival pathways [134,136]. Approaches using gemcitabine/nab-paclitaxel or FOLFIRINOX have included agents targeting DNA damage response, stromal signaling, apoptosis, and immune-cell recruitment [88,92]. Early studies involving CCR2/CXCR2, WEE1, and XPO1 inhibition demonstrated preliminary biological activity, although clinical benefits remain limited and inconsistent [88,137,138].

#### 5.3.2. Targeted Plus Immunotherapy

Combination strategies incorporating immunotherapy aim to overcome the immunosuppressive, T-cell-excluded tumor microenvironment characteristic of PDAC [135,139,140]. Investigated approaches include combining PD-1/PD-L1 blockade with PARP inhibitors in homologous recombination-deficient tumors, as well as targeting KRAS, FAK, CSF1R, CXCR4–CXCL12, and TGF-β pathways to enhance immune infiltration and reduce immune suppression [134,138,140]. Although preclinical and early clinical findings are encouraging, clinical benefit remains modest, and no phase III trial has demonstrated a clear survival advantage, highlighting the persistent challenge of overcoming the highly stromal and immunosuppressive PDAC microenvironment [88,134,135].

### 5.4. Neoadjuvant and Perioperative Targeted and Immune Strategies

Neoadjuvant systemic therapy is increasingly used for borderline-resectable PDAC and is under investigation in selected patients with resectable disease. Its potential advantages include early treatment of occult micrometastatic disease, assessment of tumor biology and treatment responsiveness, and an increased likelihood of margin-negative resection. Modified FOLFIRINOX and gemcitabine plus nab-paclitaxel remain the principal chemotherapy backbones, while the optimal treatment duration, sequencing, and contribution of radiotherapy continue to be evaluated in trials such as CASSANDRA and PREOPANC-3 [8,141,142]. Neoadjuvant molecularly targeted therapies remain at an early stage of development, with ongoing studies evaluating biomarker-selected approaches, including DNA-damage-repair and RAS-directed strategies.

Beyond molecularly targeted approaches, immune checkpoint blockade has also been evaluated in the neoadjuvant treatment of PDAC, although the available evidence remains preliminary. In a randomized phase II study involving 37 patients, the addition of pembrolizumab to neoadjuvant chemoradiotherapy was feasible but did not produce a convincing increase in CD8-positive tumor-infiltrating lymphocytes or demonstrate a clear survival benefit. Pancreatectomy was performed in 71% of patients receiving pembrolizumab and 54% of those receiving chemoradiotherapy alone; however, the study was not powered to establish differences in surgical outcomes [143]. Similarly, a single-arm phase I pilot study of modified FOLFIRINOX plus nivolumab in 28 patients with borderline-resectable PDAC reported that 22 patients (79%) proceeded to surgery, with no grade 3 or higher immune-related adverse events [144]. These studies demonstrate the feasibility of incorporating checkpoint blockade into neoadjuvant regimens but do not establish improvements in pathologic response, resection outcomes, or survival. Accordingly, perioperative checkpoint inhibition remains investigational and should be evaluated in larger randomized, biomarker-informed trials.

## 6. Molecular Profiling and Patient Selection

### 6.1. Clinical Integration of Germline and Somatic Testing

Germline and somatic testing provide complementary information in PDAC. Germline testing identifies inherited pathogenic variants, whereas somatic testing detects acquired tumor alterations relevant to targeted therapy [23,24]. Current guidelines recommend universal germline testing for all patients with PDAC because clinically significant variants, such as BRCA1/2, PALB2, ATM, CDKN2A, and mismatch repair genes may influence treatment selection and hereditary risk assessment [25,26,27]. Somatic profiling is especially important in advanced disease to identify actionable alterations including KRAS variants, NTRK fusions, BRAF alterations, ERBB2 amplification, and MSI-H/dMMR status [38,39,60]. Because tumor sequencing cannot reliably distinguish somatic from germline events, integrated germline and somatic testing is recommended to optimize targeted therapy and familial risk assessment [23,24,28,29,54].

### 6.2. Next-Generation Sequencing Panels

Somatic next-generation sequencing (NGS) panels are central to molecular profiling in PDAC because they enable simultaneous detection of mutations, copy number alterations, microsatellite instability, and gene rearrangements [24,54,145]. Compared with sequential single-gene testing, NGS provides a more comprehensive approach for identifying actionable alterations, especially in advanced disease [24,145].

Although no standard NGS panel exists for PDAC, broader DNA- and RNA-based assays are especially important in KRAS wild-type tumors, where targetable fusions such as NTRK, NRG1, RET, ALK, and ROS1 are enriched and may be missed by DNA-only testing [24,39,40,41,60]. Integrated genomic and transcriptomic profiling may further improve molecular stratification but currently remains largely limited to specialized and research settings [54,145,146].

### 6.3. Liquid Biopsy

Liquid biopsy is an emerging minimally invasive approach for molecular assessment in PDAC through analysis of circulating tumor-derived material, particularly circulating tumor DNA (ctDNA) [147,148,149,150]. It enables real-time molecular profiling and longitudinal disease monitoring, especially when tissue sampling is limited or repeat biopsies are impractical [148,149,150]. In advanced PDAC, ctDNA analysis may identify tumor alterations and monitor treatment response or emerging resistance, while postoperative ctDNA detection may indicate minimal residual disease and predict recurrence risk [149,150,151,152].

However, clinical utility remains limited by reduced sensitivity in localized or low-volume disease and variability related to tumor burden and assay methodology [147,150,153]. Alternative exosome-, CTC-, and multi-analyte-based platforms remain investigational [148,149,153].

### 6.4. Functional Precision Oncology and Drug Repurposing

Patient-derived organoids (PDOs) are three-dimensional cultures established directly from patient tumor tissue that preserve important histologic, genomic, and phenotypic characteristics of the original cancer [154,155,156]. Compared with conventional two-dimensional cell lines, PDOs more faithfully model interpatient heterogeneity and provide a functional precision oncology platform for ex vivo assessment of drug sensitivity and resistance. Unlike genomic profiling alone, organoid-based pharmacotyping offers a direct functional readout of tumor response and may capture therapeutic vulnerabilities arising from complex interactions among driver alterations, cellular lineage, epigenetic programs, and adaptive stress-response pathways.

PDOs also provide a scalable framework for drug screening and repurposing through the systematic evaluation of approved and investigational compounds. This approach may identify unexpected sensitivities to existing therapies, reveal subtype- or state-specific vulnerabilities, support biomarker discovery, and facilitate the development of rational combinations designed to overcome intrinsic or acquired resistance [156]. Recent studies have reported associations between PDO drug-response profiles and clinical treatment outcomes, supporting the potential integration of organoid-guided treatment selection into precision oncology workflows [157,158].

However, several limitations currently preclude routine clinical implementation. Standard PDO cultures incompletely reproduce stromal, vascular, and immune interactions within the PDAC tumor microenvironment. Additional challenges include variability in tissue acquisition and organoid-establishment success, differences in culture and assay methodology, the need for adequate viable tissue and specialized expertise, the scalability of high-throughput testing, and turnaround times compatible with clinical decision-making. Moreover, prospective evidence demonstrating that PDO-guided treatment selection improves patient outcomes remains limited. Standardized culture platforms, advanced co-culture models, and prospective multi-institutional clinical trials are therefore required before PDO-based pharmacotyping can be incorporated into routine practice.

### 6.5. Barriers to Implementation

Implementation of biomarker-guided therapy in PDAC remains challenging because actionable alterations are uncommon, although the emerging KRAS-targeted therapies may expand treatment opportunities for a large proportion of patients, given the high prevalence of KRAS mutations in PDAC [21,22,54]. In addition, many patients present with rapidly progressive advanced disease, limiting opportunities for molecularly guided treatment or clinical-trial enrollment [24,159]. Even when actionable alterations are identified, only a subset of patients receive matched therapies in real-world practice [54,160].

Tissue adequacy remains another major limitation, especially with small biopsy samples that may not provide sufficient material for comprehensive DNA- and RNA-based profiling [24,160,161]. Delays in testing, inconsistent guideline adherence, reimbursement issues, and limited access to molecular tumor boards further restrict implementation across healthcare systems [24,54,162,163]. Overall, broader integration of precision oncology in PDAC will require standardized testing pathways, timely molecular analysis, and improved access to biomarker-driven therapies and clinical trials [54,159,163].

## 7. Resistance Mechanisms and Limitations

### 7.1. Biological Resistance Mechanisms

Despite progress in targeted therapy for molecularly selected PDAC, durable responses remain limited because of multiple resistance mechanisms. In KRAS-directed therapy, resistance may develop through secondary KRAS alterations, KRAS amplification, or activation of bypass signaling pathways that restore downstream signaling despite initial target inhibition [62,118]. In addition, mutant KRAS drives extensive transcriptional, metabolic, and signaling rewiring programs that may become partially self-sustaining over time through secondary genomic alterations, epigenetic adaptation, and tumor microenvironmental support. Consequently, some advanced PDACs may exhibit incomplete dependence on KRAS signaling despite retaining KRAS mutations, potentially limiting the durability and depth of response to KRAS-targeted therapies. In homologous recombination-deficient tumors, PARP inhibitor resistance can occur through restoration of DNA repair pathways and broader rewiring of DNA damage response signaling [94,106].

Intratumoral heterogeneity and the dense, immunosuppressive tumor microenvironment further contribute to resistance by impairing drug delivery and sustaining pro-survival signaling [62,88,164]. Adaptive resistance mechanisms involving compensatory MAPK, PI3K, SHP2, and SOS1 pathway activation may also reduce the long-term efficacy of KRAS inhibition and provide the rationale for ongoing combination therapy strategies. These features also help explain the limited efficacy of immunotherapy outside rare MSI-H/dMMR tumors [135,165].

### 7.2. Clinical and Translational Limitations

The clinical impact of targeted therapy in PDAC remains limited because actionable alterations are uncommon, although emerging KRAS-targeted therapies may expand treatment options beyond rare subgroups such as BRCA-associated, MSI-H/dMMR, NTRK fusion-positive, and KRAS G12C-mutant tumors [56,60,62]. However, much of the available evidence derives from small subgroup analyses and basket trials rather than large pancreas-specific randomized studies [70,71,73,97].

Implementation is further limited by underutilization of molecular testing, delays in access, and tissue inadequacy for comprehensive profiling [159,163]. Together, these challenges explain why precision therapies in PDAC currently benefit only selected biomarker-defined patient subsets.

## 8. Future Directions

Future progress in targeted therapy for PDAC will build on the clinical validation of KRAS and RAS inhibition while extending precision approaches to additional molecular subgroups and earlier disease settings. Selected ongoing phase III studies and their potential clinical implications are summarized in Table 4 [100,141,142,166,167,168,169,170,171,172,173,174,175,176,177,178,179,180]. The phase III evaluation of daraxonrasib in previously treated RAS-mutant metastatic PDAC established oncogenic RAS as a clinically actionable target [93]. Ongoing studies include RASolute 303, evaluating daraxonrasib alone or with chemotherapy as first-line treatment for metastatic PDAC [174]; RASolute 304, evaluating adjuvant daraxonrasib after resection [176]; and RASolute 305, evaluating zoldonrasib plus first-line chemotherapy in metastatic KRAS G12D-mutant PDAC [100]. Important questions remain regarding durability, toxicity, acquired resistance, biomarker selection, and optimal treatment sequencing.

Further advances will require comprehensive molecular characterization and rational therapeutic development. Combined DNA- and RNA-based profiling may improve the detection of actionable sequence variants, amplifications, and gene fusions, particularly in KRAS-wild-type tumors. Distinguishing germline from somatic findings remains essential for treatment selection and hereditary risk assessment. Liquid biopsy and patient-derived organoids may complement tissue-based profiling by monitoring minimal residual disease, detecting resistance, and providing functional evidence of drug sensitivity [146,149,150,151]. Prospective studies should prioritize predictive biomarker validation, timely testing, and equitable access to molecularly guided therapies.

Overcoming adaptive resistance and the stromal and immunosuppressive PDAC microenvironment will require biomarker-informed combinations of RAS-directed therapies with chemotherapy, DNA-damage-response agents, inhibitors of compensatory signaling, immune-modulating therapies, or selective stromal and myeloid interventions. These approaches require careful evaluation of biological rationale and overlapping toxicity. Continued development of allele-specific KRAS inhibitors, multiselective RAS inhibitors, DNA-repair- and fusion-directed therapies, and functional precision-oncology strategies may broaden the clinical impact of targeted therapy across metastatic, adjuvant, and perioperative settings [54,159,163].

## 9. Conclusions

PDAC remains one of the most lethal solid malignancies, and the benefits of targeted therapy remain concentrated within selected molecularly defined subgroups. Nevertheless, PARP inhibition in selected BRCA-associated tumors, immune checkpoint blockade in MSI-H/dMMR disease, TRK inhibition in NTRK fusion-positive cancers, and multiselective RAS inhibition demonstrate the increasing clinical relevance of precision oncology in PDAC. In particular, the phase III RASolute 302 trial established the efficacy of daraxonrasib in previously treated metastatic PDAC, providing definitive clinical validation of oncogenic RAS as a therapeutic target [93].

Daraxonrasib represents a major therapeutic advance in the second-line setting, with clinically meaningful improvements in overall survival, progression-free survival, objective response, and duration of response compared with standard chemotherapy. Further investigation is required to define the durability of benefit, mechanisms of acquired resistance, optimal toxicity management, treatment sequencing, and its potential role in first-line and earlier-stage disease.

Comprehensive germline and somatic profiling, supported by integrated DNA- and RNA-based testing, remains essential for identifying actionable alterations, informing treatment selection, and facilitating enrollment in biomarker-driven clinical trials. Emerging allele-specific KRAS inhibitors, DNA-repair- and fusion-directed therapies, liquid-biopsy approaches, patient-derived organoids, and mechanistically informed combination strategies may further broaden the population benefiting from precision oncology. Continued molecular characterization, prospective biomarker validation, and rational drug development will be critical to integrating targeted therapies across multiple stages of PDAC and improving long-term clinical outcomes.

## Figures and Tables

**Table 1:** Major Molecular Events in PDAC and Their Therapeutic Relevance.

Molecular Alteration	Frequency in PDAC	Biologic & Clinical Significance	Therapeutic Relevance	Example Therapeutic Implication
KRAS (somatic sequence mutation) [21,30,34,56]	~93%	Dominant oncogenic driver; constitutive MAPK/PI3K signaling, metabolic reprogramming, and an immunosuppressive microenvironment	Major determinant of PDAC biology; direct targeting remains limited for most variants	Daraxonrasib (multiselective RAS inhibitor); allele-specific agents listed below; SHP2/SOS1-based combinations (investigational)
KRAS G12D (somatic mutation) [21,33,34,35,74,75,76,77]	~42% of KRAS-mutant PDAC	Constitutive activation of MAPK and PI3K signaling, promoting proliferation, metabolic reprogramming, and immune suppression	Associated with aggressive disease biology and poorer survival	MRTX1133; zoldonrasib (RMC-9805); ASP3082 (investigational)
KRAS G12V and other KRAS mutations ** [21,33,34,35]	KRAS G12V ~32%; others less common	Sustained oncogenic RAS signaling and therapeutic resistance	Contribute to tumor progression and heterogeneity	Daraxonrasib; other pan-KRAS/pan-RAS inhibitors and combination strategies (investigational)
KRAS G12C (somatic mutation) [23,57,58]	~1–2%	Rare actionable KRAS subtype within a predominantly KRAS-mutant disease	Directly targetable in selected patients	Sotorasib; adagrasib (tumor-type approvals exist, but PDAC use remains biomarker-directed and context dependent)
KRAS-WT subgroup (heterogeneous event classes) [38,39,40,41,60]	~7–10%	Enriched for somatic amplifications, kinase mutations, and gene fusions; individual frequencies are low	Clinically important subgroup for biomarker-guided therapy	Examples: Zenocutuzumab for eligible NRG1 fusion-positive advanced PDAC, dabrafenib plus trametinib for BRAF V600E-mutant disease, and trastuzumab deruxtecan for eligible HER2-positive (IHC 3+) tumors
TP53 (predominantly somatic mutation) [21,30,43,44,46]	~72%	Loss of genome surveillance, apoptosis dysregulation, chromosomal instability, EMT, and chemoresistance; adverse prognostic significance	Primarily biologic and prognostic relevance; no established matched therapy	Rezatapopt (PC14586) for TP53 Y220C and other p53-reactivation strategies (investigational; no established PDAC therapy)
CDKN2A (somatic mutation/deletion/methylation; rare germline variant) [21,30,46,49]	~30%	Dysregulation of G1-S cell-cycle control through CDK4/6-RB and p53-related pathways	Primarily biologic and prognostic relevance; cell-cycle targeting remains investigational	Palbociclib, ribociclib, or abemaciclib-based combinations (investigational; no established PDAC benefit)
SMAD4 (somatic mutation/deletion) [30,42,43,46]	~32%	Loss of TGF-β-mediated growth suppression; associated with metastatic dissemination and poorer outcomes	Primarily prognostic and biologic relevance; no established matched therapy	Galunisertib or other TGF-β-pathway inhibitors (investigational; not validated as SMAD4-selected therapy)
BRCA1/2 (germline or somatic pathogenic variant) [63,65,68]	Part of HRD subgroup *	Homologous recombination deficiency; platinum sensitivity and PARP inhibitor eligibility	Established biomarker-defined therapeutic relevance	Platinum-based chemotherapy; maintenance olaparib for platinum-sensitive metastatic PDAC with a germline BRCA1/2 pathogenic variant
PALB2 (germline or somatic pathogenic variant) [56,64,66]	Part of HRD subgroup *	BRCA-like homologous recombination deficiency phenotype	Emerging biomarker-defined relevance	Platinum-based chemotherapy; rucaparib or other PARP-inhibitor strategies in selected patients (evidence remains nonrandomized)
NTRK1/2/3 fusion (somatic structural rearrangement) [39,69,70]	<1%	Rare dominant fusion driver, enriched in KRAS-WT tumors	Directly actionable	Larotrectinib; entrectinib
MSI-H/dMMR (genomic phenotype; germline or somatic cause) [23,71,73]	~1–3%	Increased mutation load and neoantigen burden; immunotherapy-sensitive subgroup	Directly actionable	Pembrolizumab

* BRCA1/2 and PALB2 pathogenic variants are components of a homologous recombination deficiency subgroup present in approximately 5–10% of PDAC; BRCA2 variants are more frequent than BRCA1 or PALB2 variants. Drug examples are not equivalent to PDAC-specific regulatory approval; investigational or tumor-agnostic use is explicitly identified. **, e.g., G12R, G13D, Q61H.

**Table 2:** Selected Practice-Changing Trials and Clinically Relevant Positive Reported Cohorts.

Trial	Phase	Population & Biomarker Subgroup	Treatment Arm	Key Efficacy Result	Key Limitation	Key Finding
RASolute 302 [93]	III	Previously treated metastatic PDAC with oncogenic RAS mutations	Daraxonrasib vs. investigator’s choice chemotherapy	Median OS 13.2 vs. 6.7 months; HR 0.40; significant improvements in PFS, ORR, and duration of response	Grade ≥ 3 adverse events occurred in 61.8%; treatment-related grade ≥ 3 events in 43.6%; dose reductions in 36.1%; long-term resistance and durability remain unresolved	First phase III survival benefit with direct multiselective RAS inhibition in PDAC
POLO [63,68]	III	Metastatic PDAC with germline BRCA1/2 mutation after ≥16 weeks of first-line platinum-based chemotherapy without progression	Maintenance olaparib vs. placebo	Median PFS 7.4 vs. 3.8 months; HR 0.53 (95% CI 0.35–0.82)	No significant OS benefit on longer follow-up [68]	Established maintenance olaparib as the leading biomarker-selected targeted therapy in PDAC [63,94]
Rucaparib maintenance study [64,95]	II	Platinum-sensitive advanced PDAC with pathogenic germline or somatic BRCA1/2 or PALB2 variants	Single-arm maintenance rucaparib	Median PFS 13.1 months; OS 23.5 months	Non-randomized evidence	Supports PARP-based strategies beyond germline BRCA [56,64]
PA.3 [96]	III	Unresectable locally advanced or metastatic PDAC	Gemcitabine plus erlotinib vs. gemcitabine plus placebo	Median OS 6.24 vs. 5.91 months; HR 0.82 (95% CI 0.69–0.99) [96]	Absolute survival benefit was modest	First positive targeted trial in PDAC, but clinical impact was limited [56,96]
Larotrectinib pooled trials * [97]	I/II	NTRK fusion-positive solid tumors, including a very small PDAC subset	Single-arm larotrectinib	ORR ~79% across tumor types; pancreatic-specific benefit based on very small numbers	Pancreatic-specific efficacy estimates remain limited [97]	Supports TRK inhibition in rare fusion-positive PDAC [70,87]
Entrectinib basket studies [69,87,98]	Basket studies	NTRK fusion-positive solid tumors, including rare PDAC cases	Single-arm entrectinib	ORR 57.4% across tumor types; pancreatic cases showed partial responses or prolonged disease control	PDAC evidence extrapolated from basket studies [69]	Clinically relevant for rare fusion-positive PDAC [69,87]
KEYNOTE-158 [73]	II	Previously treated MSI-H/dMMR non-colorectal cancers, including a PDAC subset	Single-arm pembrolizumab	ORR 30.8% across MSI-H/dMMR non-colorectal tumors; pancreatic responses less frequent than in some other cancers	Very small eligible PDAC subgroup [71,72]	Supports MSI-H/dMMR as a rare but actionable biomarker in PDAC [71,72]
CodeBreaK 100 [57]	I/II	Previously treated advanced KRAS G12C-mutated PDAC	Single-arm sotorasib	ORR 21.1%; DCR 84.2% in PDAC	Restricted to a rare molecular subset [56,57]	Proof-of-concept for direct KRAS inhibition in PDAC [56,57]
RMC-GI-102 (daraxonrasib + GnP cohort) [99]	I/Ib	Previously untreated metastatic PDAC with RAS mutations	Daraxonrasib plus gemcitabine/nab-paclitaxel	High objective response rates of 58–59% with encouraging progression-free survival (84% at 6 months)	Single-arm early-phase study; immature survival data	Supports further evaluation of daraxonrasib plus first-line chemotherapy; does not establish a standard of care
KRYSTAL-1 [58]	Early-phase	KRAS G12C-mutated solid tumors, including PDAC	Single-arm adagrasib	ORR 33.3% in pancreatic cancer subgroup	Early evidence in a very small subset [56,58]	Further supports KRAS G12C as a valid but uncommon target [56,58]
RMC-GI-102 (ESMO GI 2026; 340O) [76]	I/II	Previously untreated metastatic RAS G12D-mutant PDAC	Zoldonrasib plus mFOLFIRINOX or gemcitabine/nab-paclitaxel	Efficacy-evaluable ORR 82% and DCR 96% with mFOLFIRINOX (*n* = 22); ORR 61% and DCR 90% with gemcitabine/nab-paclitaxel (*n* = 31)	Nonrandomized; efficacy evaluated in subsets of 81 enrolled patients; median follow-up 5.7–6.1 months; grade ≥ 3 TRAEs 61% and 80% [76]	Encouraging first-line activity, but no comparative evidence that zoldonrasib improves efficacy beyond chemotherapy; phase III confirmation required [76,100]
RMC-9805-001 (ESMO GI 2026; 341O) [77]	I	Previously treated KRAS G12D-mutant metastatic PDAC (2 L and ≥3 L)	Zoldonrasib plus daraxonrasib	ORR 50%, DCR 97%, median PFS 9.6 months in 2 L; ORR 47%, DCR 90%, median PFS 7.6 months and median OS 10.5 months in ≥3 L	Single-arm study with 30 patients per cohort; grade ≥ 3 TRAEs 35%; one reported treatment-related grade 5 intestinal perforation [77]	Suggests activity in heavily pretreated KRAS G12D PDAC, but contribution of each agent and comparative benefit remain unknown [77]

* Pooled larotrectinib trials included LOXO-TRK-14001, SCOUT, and NAVIGATE.

**Table 3:** Completed Trials with Negative or Limited Results that Define Current Limitations.

Trial	Phase	Population & Biomarker Subgroup	Treatment Arm	Key Efficacy Result	Key Limitation	Key Finding
CALGB 80303 [121]	III	Advanced PDAC	Gemcitabine + bevacizumab vs. gemcitabine + placebo	Median OS 5.8 vs. 5.9 months Median PFS 3.8 vs. 2.9 months	No OS benefit despite modest PFS improvement	Anti-angiogenic therapy alone was insufficient in PDAC [119,121]
AViTA [122]	III	Metastatic PDAC	Gemcitabine + erlotinib + bevacizumab vs. gemcitabine + erlotinib	Median OS 7.1 vs. 6.0 months HR 0.89 (95% CI 0.74–1.07) PFS HR 0.73 (95% CI 0.61–0.86)	No significant OS benefit	VEGF blockade did not produce meaningful survival improvement [119,122]
Selumetinib trial [123]	II	Gemcitabine-refractory advanced PDAC	Selumetinib vs. capecitabine	Median OS 5.4 vs. 5.0 months Median PFS 1.9 vs. 2.1 months ORR 0% vs. 7.1%	No improvement in response or survival	Single-agent MEK inhibition was ineffective in unselected PDAC [119,123]
Trametinib + gemcitabine trial [124]	II	Previously untreated metastatic PDAC	Trametinib + gemcitabine vs. gemcitabine alone	Median OS 8.4 vs. 6.7 months HR 0.98 (95% CI 0.67–1.44) Median PFS 16.1 vs. 15.1 weeks HR 1.08 (95% CI 0.75–1.56)	No significant improvement in OS or PFS	Indirect KRAS-pathway blockade was insufficient in unselected PDAC [119,124]
SWOG S1115 [125]	II	Metastatic PDAC after progression on gemcitabine-based therapy	Selumetinib + MK-2206 vs. modified FOLFOX	Median OS 3.9 vs. 6.7 months Median PFS 1.9 vs. 2.0 months Trial stopped early for futility	More grade ≥ 3 toxicities and treatment delays	Dual MEK/AKT inhibition did not outperform chemotherapy [125]
Everolimus study [126]	II	Gemcitabine-refractory metastatic PDAC	Single-arm everolimus	OS 4.5 months Median PFS 1.8 months No objective responses	Minimal efficacy	mTORC1 inhibition alone had minimal clinical utility in PDAC [120,126]
Everolimus + capecitabine study [127]	II	Advanced PDAC	Single-arm everolimus + capecitabine	ORR 6% Stable disease 32% Median PFS 3.6 months OS 8.9 months	Grade 3 hyperglycemia common; dose reductions frequent	Activity remained modest and toxicity limited further development [120,127]

**Table 4:** Ongoing Phase III Trials in PDAC as of July 2026 and Their Potential Clinical Impact.

NCT Number	Study Title	Status	Population & Clinical Setting	Primary Outcome and Potential Clinical Impact
NCT04793932	Short-course Versus Long-course Pre-operative Chemotherapy with mFOLFIRINOX or PAXG (CASSANDRA TRIAL) [141]	Active, not recruiting	PDAC undergoing pre-operative therapy	Event-free survival; may clarify optimal duration of preoperative multi-agent chemotherapy
NCT06608927	Study of Quemliclustat and Chemotherapy Versus Placebo and Chemotherapy in Patients with Metastatic Pancreatic Ductal Adenocarcinoma (PRISM-1) [166]	Active, not recruiting	Metastatic PDAC	Overall survival; tests whether CD73/adenosine-pathway inhibition can overcome immune suppression
NCT03899636	A Pivotal Study of Safety and Effectiveness of NanoKnife IRE for Stage 3 Pancreatic Cancer (DIRECT) [167]	Active, not recruiting	Stage III pancreatic cancer	Overall survival; evaluates local irreversible electroporation in unresectable stage III disease
NCT05314998	Adjuvant Trial in Patients with Resected PDAC Randomized to Allocation of Oxaliplatin- or Gemcitabine-based Chemotherapy by Standard Clinical Criteria or by a Transcriptomic Treatment Specific Stratification Signature [168]	Not yet recruiting	Resected PDAC; adjuvant setting	Disease-free survival; tests transcriptomic assignment of adjuvant chemotherapy
NCT07445295	Chiauranib Plus PD-1 Inhibitor, Albumin-paclitaxel and Gemcitabine in Patients with Metastatic Pancreatic Ductal Adenocarcinoma [169]	Not yet recruiting	Metastatic PDAC	Safety and overall survival; evaluates combined antiangiogenic and PD-1 pathway modulation
NCT07336953	A Phase III, Randomized, Clinical Trial of GnP Combined with SBRT and Serplulimab Versus GnP as First-Line Treatment for Patients with Recurrent or Metastatic Pancreatic Cancer (WGOG-PAN 006/ICSBR-2) [170]	Not yet recruiting	Recurrent or metastatic pancreatic cancer	Overall survival; tests chemotherapy, SBRT, and PD-1 blockade in first-line recurrent/metastatic disease
NCT07076121	A Study Comparing BMS-986504 in Combination with Nab-paclitaxel and Gemcitabine Versus Placebo in Combination with Nab-paclitaxel and Gemcitabine in Participants with Untreated Metastatic Pancreatic Ductal Adenocarcinoma with Homozygous MTAP Deletion (MountainTAP-30) [171]	Recruiting	Untreated metastatic PDAC	Progression-free and overall survival; tests a biomarker-selected MTAP-deletion strategy
NCT04969731	Safety and Efficacy of Immuncell-LC with Gemcitabine in Resectable Pancreatic Cancer [172]	Recruiting	Resectable pancreatic cancer	Recurrence-free survival; evaluates adoptive immune-cell therapy in resectable disease
NCT06079346	A Study of OT-101 with mFOLFIRINOX in Patients with Advanced and Unresectable or Metastatic Pancreatic Cancer (STOP-PC) [173]	Recruiting	Advanced unresectable or metastatic pancreatic cancer	Overall survival; tests TGF-β pathway inhibition with mFOLFIRINOX
NCT07252232	Study of Daraxonrasib (RMC-6236) in Patients with Resected Pancreatic Ductal Adenocarcinoma (PDAC) (RASolute 304) [176]	Recruiting	Resected PDAC	Disease-free survival; determines whether RAS inhibition improves outcomes after resection
NCT07165951	Clinical Trial Comparing TQB2868 Injection Combined with Anlotinib Hydrochloride Capsules with Placebo Combined with Chemotherapy as First-line Treatment for Metastatic Pancreatic Ductal Adenocarcinoma (mPDAC) [175]	Recruiting	First-line metastatic PDAC	Overall survival; tests a multi-target antiangiogenic/immunotherapy approach
NCT04927780	Perioperative or Adjuvant mFOLFIRINOX for Resectable Pancreatic Cancer (PREOPANC-3) [142]	Recruiting	Resectable pancreatic cancer; perioperative/adjuvant setting	Overall survival; may define perioperative versus adjuvant chemotherapy for resectable PDAC
NCT07491445	Study of Daraxonrasib and Daraxonrasib + GnP as First-line Treatment in Patients with Metastatic Pancreatic Adenocarcinoma (RASolute 303) [174]	Recruiting	Metastatic PDAC; first-line setting	Progression-free and overall survival; tests daraxonrasib alone or with chemotherapy in first-line metastatic disease
NCT05254171	Study of Nab-Paclitaxel and Gemcitabine with or Without SBP-101 in Pancreatic Cancer (ASPIRE) [177]	Recruiting	Metastatic/stage IV pancreatic cancer	Overall survival; evaluates polyamine-metabolism targeting with chemotherapy
NCT06783140	Study of NABPLAGEM vs. Nab-Paclitaxel/Gemcitabine in BRCA1/2 or PALB2 Pancreatic Cancer (PLATINUM-CAN) [178]	Recruiting	Advanced or metastatic BRCA1/2- or PALB2-mutated pancreatic cancer	Response and overall survival; tests platinum intensification in BRCA1/2- or PALB2-pathogenic-variant PDAC
NCT07522073	A Study to Evaluate Chemotherapy with or Without INCB161734 in Previously Untreated, KRAS G12D-Mutated Metastatic Pancreatic Ductal Adenocarcinoma (DAWN-303) [179]	Recruiting	Previously untreated KRAS G12D-mutated metastatic PDAC	Overall survival, progression-free survival, and response; may establish a KRAS G12D-directed first-line strategy
NCT06958328	Testing Higher Dose Radiation Therapy for Locally Advanced Pancreatic Cancer (LAP100) [180]	Recruiting	Locally advanced unresectable PDAC/stage III pancreatic cancer	Overall survival; tests radiation dose intensification in locally advanced PDAC
NCT07621718	Study of Zoldonrasib plus Investigator-Choice Chemotherapy versus Placebo plus Chemotherapy as First-Line Treatment in Metastatic KRAS G12D-Mutated Pancreatic Adenocarcinoma (RASolute 305) [100]	Recruiting	Previously untreated metastatic KRAS G12D-mutated pancreatic adenocarcinoma	Progression-free and overall survival; tests whether adding zoldonrasib to mFOLFIRINOX or gemcitabine/nab-paclitaxel improves first-line outcomes

Note: Studies were identified from ClinicalTrials.gov using the Phase III and active-status filters and were updated through 13 July 2026. Trial status is time sensitive; the final column explains the principal clinical question so that the table is interpreted as a research agenda rather than a registry list.

## Data Availability

No new data were created or analyzed in this study.

## References

[1] Bray F., Laversanne M., Sung H., Ferlay J., Siegel R.L., Soerjomataram I., Jemal A. (2024). Global Cancer Statistics 2022: GLOBOCAN Estimates of Incidence and Mortality Worldwide for 36 Cancers in 185 Countries. CA Cancer J. Clin..

[2] Ferlay J., Ervik M., Lam F., Laversanne M., Colombet M., Mery L., Piñeros M., Znaor A., Soerjomataram I., Bray F. (2024). Global Cancer Observatory: Cancer Today.

[3] Siegel R.L., Kratzer T.B., Wagle N.S., Sung H., Jemal A. (2026). Cancer Statistics, 2026. CA Cancer J. Clin..

[4] Rahib L., Smith B.D., Aizenberg R., Rosenzweig A.B., Fleshman J.M., Matrisian L.M. (2014). Projecting Cancer Incidence and Deaths to 2030: The Unexpected Burden of Thyroid, Liver, and Pancreas Cancers in the United States. Cancer Res..

[5] National Cancer Institute (2025). Cancer Stat Facts: Pancreatic Cancer.

[6] Brunner M., Flessa M., Jacobsen A., Merkel S., Krautz C., Weber G.F., Grützmann R. (2024). Recurrence pattern and its risk factors in patients with resected pancreatic ductal adenocarcinoma—A retrospective analysis of 272 patients. Pancreatology.

[7] Burris H.A., Moore M.J., Andersen J., Green M.R., Rothenberg M.L., Modiano M.R., Cripps M.C., Portenoy R.K., Storniolo A.M., Tarassoff P. (1997). Improvements in survival and clinical benefit with gemcitabine as first-line therapy for patients with advanced pancreas cancer: A randomized trial. J. Clin. Oncol..

[8] Conroy T., Hammel P., Hebbar M., Ben Abdelghani M., Wei A.C., Raoul J.-L., Choné L., Francois E., Artru P., Biagi J.J. (2018). FOLFIRINOX or Gemcitabine as Adjuvant Therapy for Pancreatic Cancer. N. Engl. J. Med..

[9] Von Hoff D.D., Ervin T., Arena F.P., Chiorean E.G., Infante J., Moore M., Seay T., Tjulandin S.A., Ma W.W., Saleh M.N. (2013). Increased survival in pancreatic cancer with nab-paclitaxel plus gemcitabine. N. Engl. J. Med..

[10] Wainberg Z.A., Melisi D., Macarulla T., Pazo-Cid R., Chandana S.R., De C., Dean A., Kiss I., Lee W., Goetze T.O. (2023). NALIRIFOX versus nab-paclitaxel and gemcitabine in treatment-naive patients with metastatic pancreatic ductal adenocarcinoma (NAPOLI 3): A randomised, open-label, phase 3 trial. Lancet.

[11] Conroy T., Ducreux M. (2025). ESMO Clinical Practice Guideline Express Update on the management of metastatic pancreatic cancer. ESMO Open.

[12] Sodergren M.H., Mangal N., Wasan H., Sadanandam A., Balachandran V.P., Jiao L.R., Habib N. (2020). Immunological combination treatment holds the key to improving survival in pancreatic cancer. J. Cancer Res. Clin. Oncol..

[13] Garrido-Laguna I., Hidalgo M. (2015). Pancreatic cancer: From state-of-the-art treatments to promising novel therapies. Nat. Rev. Clin. Oncol..

[14] Conroy T., Desseigne F., Ychou M., Bouché O., Guimbaud R., Bécouarn Y., Adenis A., Raoul J.-L., Gourgou-Bourgade S., de la Fouchardière C. (2011). FOLFIRINOX versus Gemcitabine for Metastatic Pancreatic Cancer. N. Engl. J. Med..

[15] Varadhachary G.R., Wolff R.A. (2016). Current and Evolving Therapies for Metastatic Pancreatic Cancer: Are We Stuck with Cytotoxic Chemotherapy?. J. Oncol. Pract..

[16] Erkan M., Reiser-Erkan C., Michalski C.W., Kleeff J. (2010). Tumor microenvironment and progression of pancreatic cancer. Exp. Oncol..

[17] Olson P., Hanahan D. (2009). Breaching the Cancer Fortress. Science.

[18] Masamune A., Shimosegawa T. (2009). Signal transduction in pancreatic stellate cells. J. Gastroenterol..

[19] Masamune A., Shimosegawa T. (2013). Pancreatic stellate cells—Multi-functional cells in the pancreas. Pancreatology.

[20] Argyriou A.A., Bruna J., Marmiroli P., Cavaletti G. (2012). Chemotherapy-induced peripheral neurotoxicity (CIPN): An update. Crit. Rev. Oncol. Hematol..

[21] Hu H., Ye Z., Qin Y., Xu X., Yu X., Zhuo Q., Ji S. (2021). Mutations in key driver genes of pancreatic cancer: Molecularly targeted therapies and other clinical implications. Acta Pharmacol. Sin..

[22] Qian Y., Gong Y., Fan Z., Luo G., Huang Q., Deng S., Cheng H., Jin K., Ni Q., Yu X. (2020). Molecular alterations and targeted therapy in pancreatic ductal adenocarcinoma. J. Hematol. Oncol..

[23] National Comprehensive Cancer Network (2025). NCCN Guidelines for Patients: Pancreatic Cancer.

[24] Zhen D.B., Safyan R.A., Konick E.Q., Nguyen R., Prichard C.C., Chiorean E.G. (2023). The role of molecular testing in pancreatic cancer. Ther. Adv. Gastroenterol..

[25] Llach J., Luzko I., Earl J., Barreto E., Rodríguez-Garrote M., Lleixà M., Herrera-Pariente C., Fernández G., Munoz J., Bonjoch L. (2024). Should We Offer Universal Germline Genetic Testing to All Patients with Pancreatic Cancer? A Multicenter Study. Cancers.

[26] Blanco Abad C., Pons P.G., Ramírez S.C., Alejandro M.Á., Torres I., Miramar D., Álvarez S.I., Marques E.P., Cid R.P. (2025). Hereditary Pancreatic Cancer: Advances in Genetic Testing, Early Detection Strategies, and Personalized Management. J. Clin. Med..

[27] Kryklyva V., Pflüger M.J., Ouchene H., Volleberg-Gorissen H., Mensenkamp A.R., Jonker M.A., van de Water C., Nagtegaal I.D., Ligtenberg M.J.L., Brosens L.A.A. (2025). Germline Pathogenic Variants in Patients with Pancreatic Ductal Adenocarcinoma and Extra-Pancreatic Malignancies: A Nationwide Database Analysis. Mod. Pathol..

[28] Tung N., Ricker C., Messersmith H., Balmaña J., Domchek S., Stoffel E.M., Almhanna K., Arun B., Chavarri-Guerra Y., Cohen S.A. (2024). Selection of Germline Genetic Testing Panels in Patients with Cancer: ASCO Guideline. J. Clin. Oncol..

[29] Stout L.A., Hunter C., Schroeder C., Kassem N., Schneider B.P. (2023). Clinically significant germline pathogenic variants are missed by tumor genomic sequencing. npj Genom. Med..

[30] Raphael B.J., Hruban R.H., Aguirre A.J., Moffitt R.A., Yeh J.J., Stewart C., Robertson A.G., Cherniack A.D., Gupta M., Getz G. (2017). Integrated Genomic Characterization of Pancreatic Ductal Adenocarcinoma (The Cancer Genome Atlas Research Network). Cancer Cell.

[31] Taherian M., Wang H., Wang H. (2022). Pancreatic Ductal Adenocarcinoma: Molecular Pathology and Predictive Biomarkers. Cells.

[32] Debernardi S., Liszka L., Ntala C., Steiger K., Esposito I., Carlotti E., Baker A., McDonald S., Graham T., Dmitrovic B. (2024). Molecular characteristics of early-onset pancreatic ductal adenocarcinoma. Mol. Oncol..

[33] Sun H., Zhang B., Li H. (2020). The Roles of Frequently Mutated Genes of Pancreatic Cancer in Regulation of Tumor Microenvironment. Technol. Cancer Res. Treat..

[34] Stickler S., Rath B., Hamilton G. (2024). Targeting KRAS in pancreatic cancer. Oncol. Res..

[35] Nusrat F., Khanna A., Jain A., Jiang W., Lavu H., Yeo C.J., Bowne W., Nevler A. (2024). The Clinical Implications of KRAS Mutations and Variant Allele Frequencies in Pancreatic Ductal Adenocarcinoma. J. Clin. Med..

[36] Kent O.A. (2018). Increased mutant KRAS gene dosage drives pancreatic cancer progression: Evidence for wild-type KRAS as a tumor suppressor?. Hepatobiliary Surg. Nutr..

[37] Varghese A.M., Perry M.A., Chou J.F., Nandakumar S., Muldoon D., Erakky A., Zucker A., Fong C., Mehine M., Nguyen B. (2025). Clinicogenomic landscape of pancreatic adenocarcinoma identifies KRAS mutant dosage as prognostic of overall survival. Nat. Med..

[38] Kumar P.A., Serinelli S., Zaccarini D.J., Huang R., Danziger N., Janovitz T., Basnet A., Sivapiragasam A., Graziano S., Ross J.S. (2023). Genomic landscape of clinically advanced KRAS wild-type pancreatic ductal adenocarcinoma. Front. Oncol..

[39] Singh H., Keller R.B., Kapner K.S., Dilly J., Raghavan S., Yuan C., Cohen E.F., Tolstorukov M., Andrews E., Brais L.K. (2023). Oncogenic Drivers and Therapeutic Vulnerabilities in KRAS Wild-Type Pancreatic Cancer. Clin. Cancer Res..

[40] Reissig T.M., Tzianopoulos I., Liffers S.-T., Rosery V.K., Guyot M., Ting S., Wiesweg M., Kasper S., Meister P., Herold T. (2023). Smaller panel, similar results: Genomic profiling and molecularly informed therapy in pancreatic cancer. ESMO Open.

[41] Fusco M.J., Saeed-Vafa D., Carballido E.M., Boyle T.A., Malafa M., Blue K.L., Teer J.K., Walko C.M., McLeod H.L., Hicks J.K. (2021). Identification of Targetable Gene Fusions and Structural Rearrangements to Foster Precision Medicine in KRAS Wild-Type Pancreatic Cancer. JCO Precis. Oncol..

[42] Yachida S., White C.M., Naito Y., Zhong Y., Brosnan J.A., Macgregor-Das A.M., Morgan R.A., Saunders T., Laheru D.A., Herman J.M. (2012). Clinical Significance of the Genetic Landscape of Pancreatic Cancer and Implications for Identification of Potential Long-term Survivors. Clin. Cancer Res..

[43] McIntyre C.A., Lawrence S.A., Richards A.L., Chou J.F., Wong W., Capanu M., Berger M.F., Donoghue M.T.A., Yu K.H., Varghese A.M. (2020). Alterations in driver genes are predictive of survival in patients with resected pancreatic ductal adenocarcinoma. Cancer.

[44] Kim M.K., Cho I.R., Kim Y., Choi J.H., Jung K., Kim J., Kim S., Yun H., Yoon J., Oh D.-Y. (2024). Prognostic value of the TP53 mutation in patients with pancreatic ductal adenocarcinoma receiving FOLFIRINOX. Ther. Adv. Med. Oncol..

[45] Saha G., Singh R., Mandal A., Das S., Chattopadhyay E., Panja P., Roy P., DeSarkar N., Gulati S., Ghatak S. (2020). A novel hotspot and rare somatic mutation p.A138V, at TP53 is associated with poor survival of pancreatic ductal and periampullary adenocarcinoma patients. Mol. Med..

[46] Stefanoudakis D., Frountzas M., Schizas D., Michalopoulos N.V., Drakaki A., Toutouzas K.G. (2024). Significance of TP53, CDKN2A, SMAD4 and KRAS in Pancreatic Cancer. Curr. Issues Mol. Biol..

[47] Hayashi A., Hong J., Iacobuzio-Donahue C.A. (2021). The pancreatic cancer genome revisited. Nat. Rev. Gastroenterol. Hepatol..

[48] Llach J., Aguilera P., Sánchez A., Ginès A., Fernández-Esparrach G., Soy G., Sendino O., Vaquero E., Carballal S., Ausania F. (2023). Pancreatic Cancer Surveillance in Carriers of a Germline Pathogenic Variant in CDKN2A. Cancers.

[49] Kimura H., Paranal R.M., Nanda N., Wood L.D., Eshleman J.R., Hruban R.H., Goggins M.G., Klein A.P., Roberts N.J. (2022). Functional CDKN2A assay identifies frequent deleterious alleles misclassified as variants of uncertain significance. eLife.

[50] Kawanaka Y., Inagaki C., Okura M., Mitani S., Takahama T., Yonesaka K., Chiba Y., Nakagawa K., Kawakami H., Hayashi H. (2025). Prognostic impact of gene alterations via homologous recombination DNA repair gene alteration status in pancreatic ductal adenocarcinoma. Front. Med..

[51] Stoffel E.M., Brand R.E., Goggins M. (2023). Pancreatic Cancer: Changing Epidemiology and New Approaches to Risk Assessment, Early Detection, and Prevention. Gastroenterology.

[52] Cazacu I.M., Farkas N., Garami A., Balaskó M., Mosdósi B., Alizadeh H., Gyöngyi Z., Rakonczay Z., Vigh É., Habon T. (2018). Pancreatitis-Associated Genes and Pancreatic Cancer Risk: A Systematic Review and Meta-Analysis. Pancreas.

[53] Pishvaian M.J., Blais E.M., Brody J.R., Lyons E., DeArbeloa P., Hendifar A., Mikhail S., Chung V., Sahai V., Sohal D.P.S. (2020). Overall survival in patients with pancreatic cancer receiving matched therapies following molecular profiling: A retrospective analysis of the Know Your Tumor registry trial. Lancet Oncol..

[54] Tarabay A., Swales L., Smolenschi C., Akoury E., Valéry M., Fuerea A., Pudlarz T., Boige V., Rouleau E., Gelli M. (2025). Precision medicine strategy in pancreatic ductal adenocarcinoma. ESMO Open.

[55] Liang K., Guo M.Z., Zaidi N. (2025). Emerging Systemic Therapy Considerations for Pancreatic Cancer. Semin. Radiat. Oncol..

[56] Fivaz M., Bornand A., Corro C., Koessler T., Genoud V. (2025). Actionable mutations in pancreatic cancer: Where targeted therapies are making a difference. BMJ Open Gastroenterol..

[57] Strickler J.H., Satake H., George T.J., Yaeger R., Hollebecque A., Garrido-Laguna I., Schuler M., Burns T.F., Coveler A.L., Falchook G.S. (2023). Sotorasib in KRAS p.G12C–Mutated advanced pancreatic cancer. N. Engl. J. Med..

[58] Bekaii-Saab T.S., Yaeger R., Spira A.I., Pelster M., Sabari J.K., Hafez N., Barve M., Velastegui K., Yan X., Shetty A. (2023). Adagrasib in Advanced Solid Tumors Harboring a KRAS^G12C^ Mutation. J. Clin. Oncol..

[59] Pradeep R., Isbeih N.J., Abraham F.F., Noori E., Yeung Z.P., Kundranda M.N. (2026). KRAS Inhibition in Pancreatic Ductal Adenocarcinoma. J. Clin. Med..

[60] Krupa K., Fudalej M., Miski H., Włoszek E., Szymczak M., Badowska-Kozakiewicz A., Czerw A., Deptąła A. (2025). KRAS-Wild Pancreatic Cancer—More Targets than Treatment Possibilities?. Cancers.

[61] Singhal A., Li B.T., O’Reilly E.M. (2024). Targeting KRAS in cancer. Nat. Med..

[62] Drizyte-Miller K., Talabi T., Somasundaram A., Cox A.D., Der C.J. (2025). KRAS: The Achilles’ heel of pancreas cancer biology. J. Clin. Investig..

[63] Golan T., Hammel P., Reni M., Van Cutsem E., Macarulla T., Hall M.J., Park J.-O., Hochhauser D., Arnold D., Oh D.-Y. (2019). Maintenance Olaparib for Germline BRCA-Mutated Metastatic Pancreatic Cancer. N. Engl. J. Med..

[64] Reiss K.A., Mick R., O’Hara M.H., Teitelbaum U., Karasic T.B., Schneider C., Cowden S., Southwell T., Romeo J., Izgur N. (2021). Phase II Study of Maintenance Rucaparib in Patients with Platinum-Sensitive Advanced Pancreatic Cancer and a Pathogenic Germline or Somatic Variant in BRCA1, BRCA2, or PALB2. J. Clin. Oncol..

[65] Wattenberg M.M., Asch D., Yu S., O’Dwyer P.J., Domchek S.M., Nathanson K.L., Rosen M.A., Beatty G.L., Siegelman E.S., Reiss K.A. (2020). Platinum response characteristics of patients with pancreatic ductal adenocarcinoma and a germline BRCA1, BRCA2 or PALB2 mutation. Br. J. Cancer.

[66] Perkhofer L., Gout J., Roger E., Kude de Almeida F., Baptista Simões C., Wiesmüller L., Seufferlein T., Kleger A. (2021). DNA damage repair as a target in pancreatic cancer: State-of-the-art and future perspectives. Gut.

[67] Pishvaian M.J., Blais E.M., Brody J.R., Rahib L., Lyons E., De Arbeloa P., Hendifar A., Mikhail S., Chung V., Sohal D.P.S. (2019). Outcomes in Patients with Pancreatic Adenocarcinoma with Genetic Mutations in DNA Damage Response Pathways: Results from the Know Your Tumor Program. JCO Precis. Oncol..

[68] Kindler H.L., Hammel P., Reni M., Van Cutsem E., Macarulla T., Hall M.J., Park J.O., Hochhauser D., Arnold D., Oh D.-Y. (2022). Overall Survival Results from the POLO Trial: A Phase III Study of Active Maintenance Olaparib Versus Placebo for Germline BRCA-Mutated Metastatic Pancreatic Cancer. J. Clin. Oncol..

[69] Demetri G.D., De Braud F., Drilon A., Siena S., Patel M.R., Cho B.C., Liu S.V., Ahn M.-J., Chiu C.-H., Lin J.J. (2022). Updated Integrated Analysis of the Efficacy and Safety of Entrectinib in Patients with NTRK Fusion-Positive Solid Tumors. Clin. Cancer Res..

[70] Drilon A., Laetsch T.W., Kummar S., DuBois S.G., Lassen U.N., Demetri G.D., Nathenson M., Doebele R.C., Farago A.F., Pappo A.S. (2018). Efficacy of Larotrectinib in TRK Fusion–Positive Cancers in Adults and Children. N. Engl. J. Med..

[71] Marabelle A., Le D.T., Ascierto P.A., Di Giacomo A.M., De Jesus-Acosta A., Delord J.-P., Geva R., Gottfried M., Penel N., Hansen A.R. (2020). Efficacy of Pembrolizumab in Patients with Noncolorectal High Microsatellite Instability/Mismatch Repair–Deficient Cancer: Results from the Phase II KEYNOTE-158 Study. J. Clin. Oncol..

[72] Dai M., Sheng J., Zhang Q., Wang J., Fu Q., Liang T. (2023). Case Report: Partial response to single-agent pembrolizumab in a chemotherapy-resistant metastatic pancreatic cancer patient with a high tumor mutation burden. Front. Oncol..

[73] Maio M., Ascierto P.A., Manzyuk L., Motola-Kuba D., Penel N., Cassier P.A., Bariani G.M., De Jesus Acosta A., Doi T., Longo F. (2022). Pembrolizumab in microsatellite instability high or mismatch repair deficient cancers: Updated analysis from the phase II KEYNOTE-158 study. Ann. Oncol..

[74] Wei D., Wang L., Zuo X., Maitra A., Bresalier R.S. (2024). A Small Molecule with Big Impact: MRTX1133 Targets the KRASG12D Mutation in Pancreatic Cancer. Clin. Cancer Res..

[75] Yoshinari T., Nagashima T., Ishioka H., Inamura K., Nishizono Y., Tasaki M., Iguchi K., Suzuki A., Sato C., Nakayama A. (2025). Discovery of KRAS(G12D) selective degrader ASP3082. Commun. Chem..

[76] Wolpin B.M., Pant S., Spira A., Biachi de Castria T., Botta G.P., Florou V., Manji G., Opsipov A., Pelster M., Sen S. Safety and Efficacy of Zoldonrasib (RMC-9805) plus Chemotherapy in Patients with First-Line RAS G12D Metastatic Pancreatic Adenocarcinoma. Proceedings of the ESMO Gastrointestinal Cancers Congress 2026.

[77] Azad N.S., Kim D., Oberstein P., Mannion B., Pelster M., Ng K., Yaeger R., Kim E.J.-H., Papadopoulos K., Lorusso P.M. Safety and Efficacy of Zoldonrasib (RMC-9805) plus Daraxonrasib (RMC-6236) in Patients with Second-Line or Later KRAS G12D Metastatic Pancreatic Adenocarcinoma. Proceedings of the ESMO Gastrointestinal Cancers Congress 2026.

[78] Chabner B.A., Roberts T.G. (2005). Timeline: Chemotherapy and the war on cancer. Nat. Rev. Cancer.

[79] Wujcik D. (2014). Science and Mechanism of Action of Targeted Therapies in Cancer Treatment. Semin. Oncol. Nurs..

[80] Abbott M., Ustoyev Y. (2019). Cancer and the Immune System: The History and Background of Immunotherapy. Semin. Oncol. Nurs..

[81] Bailey P., Chang D.K., Nones K., Johns A.L., Patch A.-M., Gingras M.-C., Miller D.K., Christ A.N., Bruxner T.J.C., Quinn M.C. (2016). Genomic analyses identify molecular subtypes of pancreatic cancer. Nature.

[82] Waddell N., Pajic M., Patch A.-M., Chang D.K., Kassahn K.S., Bailey P., Johns A.L., Miller D., Nones K., Quek K. (2015). Whole genomes redefine the mutational landscape of pancreatic cancer. Nature.

[83] Moffitt R.A., Marayati R., Flate E.L., Volmar K.E., Loeza S.G., Hoadley K.A., Rashid N.U., Williams L.A., Eaton S.C., Chung A.H. (2015). Virtual microdissection identifies distinct tumor- and stroma-specific subtypes of pancreatic ductal adenocarcinoma. Nat. Genet..

[84] Collisson E.A., Sadanandam A., Olson P., Gibb W.J., Truitt M., Gu S., Cooc J., Weinkle J., Kim G.E., Jakkula L. (2011). Subtypes of pancreatic ductal adenocarcinoma and their differing responses to therapy. Nat. Med..

[85] Shaya J., Kato S., Adashek J.J., Patel H., Fanta P.T., Botta G.P., Sicklick J.K., Kurzrock R. (2023). Personalized matched targeted therapy in advanced pancreatic cancer: A pilot cohort analysis. npj Genom. Med..

[86] Conroy T., Pfeiffer P., Vilgrain V., Lamarca A., Seufferlein T., O’Reilly E.M., Hackert T., Golan T., Prager G.W., Haustermans K. (2023). Pancreatic cancer: ESMO Clinical Practice Guideline for diagnosis, treatment and follow-up. Ann. Oncol..

[87] Li B., Zhang Q., Castaneda C., Cook S. (2024). Targeted Therapies in Pancreatic Cancer: A New Era of Precision Medicine. Biomedicines.

[88] Rémond M.S., Pellat A., Brezault C., Dhooge M., Coriat R. (2022). Are targeted therapies or immunotherapies effective in metastatic pancreatic adenocarcinoma?. ESMO Open.

[89] Manji G.A., Olive K.P., Saenger Y.M., Oberstein P. (2017). Current and Emerging Therapies in Metastatic Pancreatic Cancer. Clin. Cancer Res..

[90] Uzunparmak B., Sahin I.H. (2019). Pancreatic cancer microenvironment: A current dilemma. Clin. Transl. Med..

[91] Deiana C., Agostini M., Brandi G., Giovannetti E. (2024). The trend toward more target therapy in pancreatic ductal adenocarcinoma. Expert Rev. Anticancer Ther..

[92] Leroux C., Konstantinidou G. (2021). Targeted Therapies for Pancreatic Cancer: Overview of Current Treatments and New Opportunities for Personalized Oncology. Cancers.

[93] O’Reilly E.M., Wainberg Z.A., Hendifar A.E., Borad M.J., Pietrantonio F., Pant S., Hammel P., Cremolini C., Manji G.A., Oberstein P.E. (2026). Daraxonrasib or chemotherapy in previously treated metastatic pancreatic cancer. N. Engl. J. Med..

[94] Anbil S., Reiss K.A. (2024). Targeting BRCA and PALB2 in Pancreatic Cancer. Curr. Treat. Options Oncol..

[95] Reiss K.A., O’Hara M.H., Teitelbaum U.R., Karasic T.B., Schneider C.J., Pratz C.F., Nathanson K.L., Vonderheide R.H., Domchek S.M. (2024). A phase II study of maintenance rucaparib in patients with platinum sensitive, advanced pancreatic cancer and a pathogenic germline or somatic variant in BRCA1, BRCA2 or PALB2: A four year survival update. J. Clin. Oncol..

[96] Moore M.J., Goldstein D., Hamm J., Figer A., Hecht J.R., Gallinger S., Au H.J., Murawa P., Walde D., Wolff R.A. (2007). Erlotinib Plus Gemcitabine Compared with Gemcitabine Alone in Patients with Advanced Pancreatic Cancer: A Phase III Trial of the National Cancer Institute of Canada Clinical Trials Group. J. Clin. Oncol..

[97] Hong D.S., DuBois S.G., Kummar S., Farago A.F., Albert C.M., Rohrberg K.S., van Tilburg C.M., Nagasubramanian R., Berlin J.D., Federman N. (2020). Larotrectinib in patients with TRK fusion-positive solid tumours: A pooled analysis of three phase 1/2 clinical trials. Lancet Oncol..

[98] Doebele R.C., Drilon A., Paz-Ares L., Siena S., Shaw A.T., Farago A.F., Blakely C.M., Seto T., Cho B.C., Tosi D. (2020). Entrectinib in patients with advanced or metastatic NTRK fusion-positive solid tumours: Integrated analysis of three phase 1–2 trials. Lancet Oncol..

[99] Wolpin B.M., Musher B.L., Manji G.A., Park W., Spira A., Azad N., Florou V., De Castria T.B., O’Hara M.H., Borazanci E. Daraxonrasib plus chemotherapy as first-line treatment for patients with metastatic pancreatic adenocarcinoma. Proceedings of the 2026 AACR Annual Meeting.

[100] National Library of Medicine (2026). Study of Zoldonrasib Plus Investigator’s-Choice Chemotherapy Versus Placebo Plus Investigator’s-Choice Chemotherapy as First-Line Treatment in Metastatic KRAS G12D-Mutated Pancreatic Adenocarcinoma (RASolute 305).

[101] Principe D.R. (2022). Precision Medicine for BRCA/PALB2-Mutated Pancreatic Cancer and Emerging Strategies to Improve Therapeutic Responses to PARP Inhibition. Cancers.

[102] Crowley F., Park W., O’Reilly E.M. (2021). Targeting DNA damage repair pathways in pancreas cancer. Cancer Metastasis Rev..

[103] Casolino R., Paiella S., Azzolina D., Beer P.A., Corbo V., Lorenzoni G., Gregori D., Golan T., Braconi C., Froeling F.E.M. (2021). Homologous Recombination Deficiency in Pancreatic Cancer: A Systematic Review and Prevalence Meta-Analysis. J. Clin. Oncol..

[104] LaRose M., Manji G.A., Bates S.E. (2024). Beyond BRCA: Diagnosis and management of homologous recombination repair deficient pancreatic cancer. Semin. Oncol..

[105] Blair A.B., Groot V.P., Gemenetzis G., Wei J., Cameron J.L., Weiss M.J., Goggins M., Wolfgang C.L., Yu J., He J. (2018). BRCA1/BRCA2 Germline Mutation Carriers and Sporadic Pancreatic Ductal Adenocarcinoma. J. Am. Coll. Surg..

[106] Mekonnen N., Yang H., Shin Y.K. (2022). Homologous Recombination Deficiency in Ovarian, Breast, Colorectal, Pancreatic, Non-Small Cell Lung and Prostate Cancers, and the Mechanisms of Resistance to PARP Inhibitors. Front. Oncol..

[107] Kindler H.L., Hammel P., Reni M., Cutsem E.V., Mercade T.M., Hall M.J., Park J.O., Hochhauser D., Arnold D., Oh D.-Y. (2019). Olaparib as maintenance treatment following first-line platinum-based chemotherapy (PBC) in patients (pts) with a germline BRCA mutation and metastatic pancreatic cancer (mPC): Phase III POLO trial. J. Clin. Oncol..

[108] Yang Z.-Y., Yuan J.-Q., Di M.-Y., Zheng D.-Y., Chen J.-Z., Ding H., Wu X.-Y., Huang Y.-F., Mao C., Tang J.-L. (2013). Gemcitabine plus erlotinib for advanced pancreatic cancer: A systematic review with meta-analysis. PLoS ONE.

[109] Liu X.-Y., Pan H.-N., Yu Y. (2024). Clinical efficacy and safety of erlotinib combined with chemotherapy in the treatment of advanced pancreatic cancer: A meta-analysis. World J. Gastrointest. Surg..

[110] Sohal D.P.S., Mangu P.B., Khorana A.A., Shah M.A., Philip P.A., O’Reilly E.M., Uronis H.E., Ramanathan R.K., Crane C.H., Engebretson A. (2016). Metastatic Pancreatic Cancer: American Society of Clinical Oncology Clinical Practice Guideline. J. Clin. Oncol..

[111] O’Reilly E.M., Hechtman J.F. (2019). Tumour response to TRK inhibition in a patient with pancreatic adenocarcinoma harbouring an NTRK gene fusion. Ann. Oncol..

[112] Yoshino T., Pentheroudakis G., Mishima S., Overman M.J., Yeh K.-H., Baba E., Naito Y., Calvo F., Saxena A., Chen L.-T. (2020). JSCO–ESMO–ASCO–JSMO–TOS: International expert consensus recommendations for tumour-agnostic treatments in patients with solid tumours with microsatellite instability or NTRK fusions. Ann. Oncol..

[113] Moore A.R., Rosenberg S.C., McCormick F., Malek S. (2020). RAS-targeted therapies: Is the undruggable drugged?. Nat. Rev. Drug Discov..

[114] Jiang J., Jiang L., Maldonato B.J., Wang Y., Holderfield M., Aronchik I., Winters I.P., Salman Z., Blaj C., Menard M. (2024). Translational and Therapeutic Evaluation of RAS-GTP Inhibition by RMC-6236 in RAS-Driven Cancers. Cancer Discov..

[115] Arbour K.C., Pubnkar S.R., Garrido-Laguna I., Hong D.S., Wolpin B., Pelster M.S., Barve M., Starodub A., Sommerhalder D., Chang S. Preliminary clinical activity of RMC-6236, a first-in-class RAS(ON) multi-selective tri-complex inhibitor, in patients with RAS-mutant solid tumors. Proceedings of the ESMO Congress 2023.

[116] O’Reilly E.M., Wolpin B., Pant S., Hecht J.R., Valerin J., Kim D.W., Azad N., Aung K., Tao L., Kar S. Daraxonrasib monotherapy as first-line treatment for patients with metastatic pancreatic adenocarcinoma. Proceedings of the 2026 AACR Annual Meeting.

[117] Ryan M.B., Corcoran R.B. (2018). Therapeutic strategies to target RAS-mutant cancers. Nat. Rev. Clin. Oncol..

[118] Dilly J., Hoffman M.T., Abbassi L., Li Z., Paradiso F., Parent B.D., Hennessey C.J., Jordan A.C., Morgado M., Dasgupta S. (2024). Mechanisms of resistance to oncogenic KRAS inhibition in pancreatic cancer. Cancer Discov..

[119] Garcia-Sampedro A., Gaggia G., Ney A., Mahamed I., Acedo P. (2021). The State-of-the-Art of Phase II/III Clinical Trials for Targeted Pancreatic Cancer Therapies. J. Clin. Med..

[120] Babiker H.M., Karass M., Recio-Boiles A., Chandana S.R., McBride A., Mahadevan D. (2019). Everolimus for the treatment of advanced pancreatic ductal adenocarcinoma (PDAC). Expert Opin. Investig. Drugs.

[121] Kindler H.L., Niedzwiecki D., Hollis D., Sutherland S., Schrag D., Hurwitz H., Innocenti F., Mulcahy M.F., O’Reilly E., Wozniak T.F. (2010). Gemcitabine Plus Bevacizumab Compared with Gemcitabine Plus Placebo in Patients with Advanced Pancreatic Cancer: Phase III Trial of the Cancer and Leukemia Group B (CALGB 80303). J. Clin. Oncol..

[122] Van Cutsem E., Vervenne W.L., Bennouna J., Humblet Y., Gill S., Van Laethem J.-L., Verslype C., Scheithauer W., Shang A., Cosaert J. (2009). Phase III Trial of Bevacizumab in Combination with Gemcitabine and Erlotinib in Patients with Metastatic Pancreatic Cancer. J. Clin. Oncol..

[123] Bodoky G., Timcheva C., Spigel D.R., La Stella P.J., Ciuleanu T.E., Pover G., Tebbutt N.C. (2012). A phase II open-label randomized study to assess the efficacy and safety of selumetinib (AZD6244 [ARRY-142886]) versus capecitabine in patients with advanced or metastatic pancreatic cancer who have failed first-line gemcitabine therapy. Investig. New Drugs.

[124] Infante J.R., Somer B.G., Park J.O., Li C.-P., Scheulen M.E., Kasubhai S.M., Oh D.-Y., Liu Y., Redhu S., Steplewski K. (2014). A randomised, double-blind, placebo-controlled trial of trametinib, an oral MEK inhibitor, in combination with gemcitabine for patients with untreated metastatic adenocarcinoma of the pancreas. Eur. J. Cancer.

[125] Chung V., McDonough S., Philip P.A., Cardin D., Wang-Gillam A., Hui L., Tejani M.A., Seery T.E., Dy I.A., Al Baghdadi T. (2017). Effect of Selumetinib and MK-2206 vs. Oxaliplatin and Fluorouracil in Patients with Metastatic Pancreatic Cancer After Prior Therapy: SWOG S1115 Study Randomized Clinical Trial. JAMA Oncol..

[126] Wolpin B.M., Hezel A.F., Abrams T., Blaszkowsky L.S., Meyerhardt J.A., Chan J.A., Enzinger P.C., Allen B., Clark J.W., Ryan D.P. (2009). Oral mTOR Inhibitor Everolimus in Patients with Gemcitabine-Refractory Metastatic Pancreatic Cancer. J. Clin. Oncol..

[127] Kordes S., Klümpen H.J., Weterman M.J., Schellens J.H.M., Richel D.J., Wilmink J.W. (2015). Phase II study of capecitabine and the oral mTOR inhibitor everolimus in patients with advanced pancreatic cancer. Cancer Chemother. Pharmacol..

[128] Hingorani S.R., Zheng L., Bullock A.J., Seery T.E., Harris W.P., Sigal D.S., Braiteh F., Ritch P.S., Zalupski M.M., Bahary N. (2018). HALO 202: Randomized Phase II Study of PEGPH20 Plus Nab-Paclitaxel/Gemcitabine Versus Nab-Paclitaxel/Gemcitabine in Patients with Untreated, Metastatic Pancreatic Ductal Adenocarcinoma. J. Clin. Oncol..

[129] Van Cutsem E., Tempero M.A., Sigal D., Oh D.Y., Fazio N., Macarulla T., Hitre E., Hammel P., Hendifar A.E., Bates S.E. (2020). Randomized Phase III Trial of Pegvorhyaluronidase Alfa with Nab-Paclitaxel Plus Gemcitabine for Patients with Hyaluronan-High Metastatic Pancreatic Adenocarcinoma. J. Clin. Oncol..

[130] Noel M., O’Reilly E.M., Wolpin B.M., Ryan D.P., Bullock A.J., Britten C.D., Linehan D.C., Belt B.A., Gamelin E.C., Ganguly B. (2020). Phase 1b study of a small molecule antagonist of human chemokine (C-C motif) receptor 2 (PF-04136309) in combination with nab-paclitaxel/gemcitabine in first-line treatment of metastatic pancreatic ductal adenocarcinoma. Investig. New Drugs.

[131] Hecht J.R., Lonardi S., Bendell J.C., Sim H.-W., Macarulla T., Lopez C.D., Van Cutsem E., Muñoz Martín A.J., Park J.O., Greil R. (2021). Randomized Phase III Study of FOLFOX Alone or with Pegilodecakin as Second-Line Therapy in Metastatic Pancreatic Cancer (SEQUOIA). J. Clin. Oncol..

[132] Smith J.P., Nkulikiyimana G.C., Cao H., Chen W., Kallakury B., Kwagyan J., Duttargi A., Weinberg B.A. (2026). Effects of Proglumide with Chemotherapy on the Pancreatic Tumor Microenvironment: Phase 1 PROGEM Trial. Pharmaceutics.

[133] Halsall J.A., Turner B.M. (2016). Histone deacetylase inhibitors for cancer therapy: An evolutionarily ancient resistance response may explain their limited success. BioEssays.

[134] Hosein A.N., Dougan S.K., Aguirre A.J., Maitra A. (2022). Translational advances in pancreatic ductal adenocarcinoma therapy. Nat. Cancer.

[135] Liu L., Huang X., Shi F., Song J., Guo C., Yang J., Liang T., Bai X. (2022). Combination therapy for pancreatic cancer: Anti-PD-(L)1-based strategy. J. Exp. Clin. Cancer Res..

[136] Singh R.R., O’Reilly E.M. (2020). New Treatment Strategies for Metastatic Pancreatic Ductal Adenocarcinoma. Drugs.

[137] Nywening T.M., Belt B.A., Cullinan D.R., Panni R.Z., Han B.J., Sanford D.E., Jacobs R., Ye J., Patel A., Gillanders W.E. (2018). Targeting both tumour-associated CXCR2+ neutrophils and CCR2+ macrophages disrupts myeloid recruitment and improves chemotherapeutic responses in pancreatic ductal adenocarcinoma. Gut.

[138] Ho W.J., Jaffee E.M., Zheng L. (2020). The tumour microenvironment in pancreatic cancer—Clinical challenges and opportunities. Nat. Rev. Clin. Oncol..

[139] Bear A.S., Vonderheide R.H., O’Hara M.H. (2020). Challenges and Opportunities for Pancreatic Cancer Immunotherapy. Cancer Cell.

[140] Fan J., Wang M.-F., Chen H.-L., Shang D., Das J.K., Song J. (2020). Current advances and outlooks in immunotherapy for pancreatic ductal adenocarcinoma. Mol. Cancer.

[141] National Library of Medicine (2021). Short-Course Versus Long-Course Pre-Operative Chemotherapy with mFOLFIRINOX or PAXG (CASSANDRA TRIAL).

[142] National Library of Medicine (2021). Perioperative or Adjuvant mFOLFIRINOX for Resectable Pancreatic Cancer (PREOPANC-3).

[143] Katz M.H.G., Petroni G.R., Bauer T., Reilley M.J., Wolpin B.M., Stucky C.C., Bekaii-Saab T.S., Elias R., Merchant N., Dias Costa A. (2023). Multicenter Randomized Controlled Trial of Neoadjuvant Chemoradiotherapy Alone or in Combination with Pembrolizumab in Patients with Resectable or Borderline-Resectable Pancreatic Adenocarcinoma. J. Immunother. Cancer.

[144] Wainberg Z.A., Link J.M., Premji A., Zheng S., Srienc M., Hammons M., Kim S.E., Li L., Liu Z., Tsvetkova O. (2026). Neoadjuvant Modified FOLFIRINOX plus Nivolumab in Borderline-Resectable Pancreatic Ductal Adenocarcinoma: A Pilot Phase 1 Trial. Nat. Commun..

[145] Hussung S., Akhoundova D., Pistoni C., Lenggenhager D., Töpfer A., Pauli C., Pestalozzi B., Britschgi C., Zoche M., Rechsteiner M. (2025). A stratified two-stage tumor molecular profiling algorithm to identify clinically actionable molecular alterations in pancreatic cancer. ESMO Gastrointest. Oncol..

[146] Knox J.J., Jang G.H., Grant R.C., Zhang A., Ma L., Elimova E., Jang R., Moore M., Biagi J., Tehfe M. (2025). Whole genome and transcriptome profiling in advanced pancreatic cancer patients on the COMPASS trial. Nat. Commun..

[147] Dubrovsky G., Ross A., Jalali P., Lotze M. (2024). Liquid Biopsy in Pancreatic Ductal Adenocarcinoma: A Review of Methods and Applications. Int. J. Mol. Sci..

[148] Bendari A., Vele O., Baskovich B., Bendari A., Sebika M., Gomez Marti J.L., Krishnamurthy K., Asiry S. (2025). Liquid Biopsy in Pancreatic Ductal Adenocarcinoma: Clinical Utility, Trials, and Future Directions. Gastroenterol. Insights.

[149] Cox M., Vitello D., Chawla A. (2025). Translating the multifaceted use of liquid biopsy to management of early disease in pancreatic adenocarcinoma. Front. Oncol..

[150] Ojha S., Sessions W., Zhou Y., Aung K.L. (2025). ctDNA in Pancreatic Adenocarcinoma: A Critical Appraisal. Curr. Oncol..

[151] Mahadevia H., Majeed U., Patel J., Ahmed A.K., Elhariri A., Albelal D., Rao N.N.M., Rachamala H.K., Mosalem O., Mukhopadhyay D. (2025). Circulating Tumor DNA and Tissue Testing for Pancreatobiliary Tumors. JAMA Netw. Open.

[152] Labori K.J. (2025). Prognostic and monitoring potential of circulating tumor DNA in resectable pancreatic cancer. Transl. Gastroenterol. Hepatol..

[153] Juthani R., Manne A. (2025). Blood-based biomarkers in pancreatic ductal adenocarcinoma: Developments over the last decade and what holds for the future—A review. Front. Oncol..

[154] Tiriac H., Belleau P., Engle D.D., Plenker D., Deschênes A., Somerville T.D.D., Froeling F.E.M., Burkhart R.A., Denroche R.E., Jang G.H. (2018). Organoid Profiling Identifies Common Responders to Chemotherapy in Pancreatic Cancer. Cancer Discov..

[155] Tiriac H., Plenker D., Baker L.A., Tuveson D.A. (2019). Organoid models for translational pancreatic cancer research. Curr. Opin. Genet. Dev..

[156] Dong H., Vizeacoumar F.S., Smith J., Jette N., Price J.D.W., Maranda V., Gong L., Freywald T., Vizeacoumar J.P., Lazell-Wright M. (2025). Organoid pharmacotyping of pancreatic cancer enables functional precision oncology and drug repurposing. bioRxiv.

[157] Grossman J.E., Muthuswamy L., Huang L., Akshinthala D., Perea S., Gonzalez R.S., Tsai L.L., Cohen J., Bockorny B., Bullock A.J. (2022). Organoid Sensitivity Correlates with Therapeutic Response in Patients with Pancreatic Cancer. Clin. Cancer Res..

[158] Beutel A.K., Ekizce M., Ettrich T.J., Seufferlein T., Lindenmayer J., Gout J., Kleger A. (2025). Organoid-based precision medicine in pancreatic cancer. United Eur. Gastroenterol. J..

[159] Jhala N., Petersen J., Jhala D. (2024). Molecular Tests in Pancreatic Cancer: Critical Role of Molecular Testing, Expanding Access, and Adherence to the NCCN Guidelines for Pancreatic Cancer. J. Natl. Compr. Cancer Netw..

[160] Strijk G.J., van Dongen J.C., de Koning W., Brosens L.A.A., Cirkel G.A., Doukas M., Sarasqueta A.F., Groenendijk F.H., Bas P., de Hingh I.H.T. (2025). Clinical relevance of next-generation sequencing in patients aged 60 years or younger with pancreatic cancer: A Nationwide prospective cohort study. Eur. J. Cancer.

[161] Pan Y., Ran T., Zhang X., Qin X., Zhang Y., Zhou C., Zou D. (2024). Adequacy of EUS–guided fine-needle aspiration and fine-needle biopsy for next-generation sequencing in pancreatic malignancies: A systematic review and meta-analysis. Endosc. Ultrasound.

[162] Petersen J.M., Jhala D.N. (2023). Compliance with the Current NCCN Guidelines and Its Critical Role in Pancreatic Adenocarcinoma. Lab. Med..

[163] Snow S., Brezden-Masley C., Carter M.D., Dhani N., Macaulay C., Ramjeesingh R., Raphael M.J., D’Angelo M.S., Servidio-Italiano F. (2024). Barriers and Unequal Access to Timely Molecular Testing Results: Addressing the Inequities in Cancer Care Delays across Canada. Curr. Oncol..

[164] Snyder G.G., Clay D., Karley S., Pipito S., Mueller R., Bradbury A., Maxwell K., Nathanson K.L., Rohanizadegan M., Shah P. (2025). Assessment of barriers to pancreatic cancer surveillance in high-risk individuals. J. Genet. Couns..

[165] Ju Y., Xu D., Liao M.-M., Sun Y., Bao W.-D., Yao F., Ma L. (2024). Barriers and opportunities in pancreatic cancer immunotherapy. npj Precis. Oncol..

[166] National Library of Medicine (2024). Study of Quemliclustat and Chemotherapy Versus Placebo and Chemotherapy in Patients with Metastatic Pancreatic Ductal Adenocarcinoma (PRISM-1).

[167] National Library of Medicine (2019). A Pivotal Study of Safety and Effectiveness of NanoKnife IRE for Stage 3 Pancreatic Cancer (DIRECT).

[168] National Library of Medicine (2022). Adjuvant Trial in Patients with Resected PDAC Randomized to Allocation of Oxaliplatin- or Gemcitabine-based Chemotherapy by Standard Clinical Criteria or by a Transcriptomic Treatment Specific Stratification Signature.

[169] National Library of Medicine (2026). Chiauranib Plus PD-1 Inhibitor, Albumin-Paclitaxel and Gemcitabine in Patients with Metastatic Pancreatic Ductal Adenocarcinoma.

[170] National Library of Medicine (2026). A Phase III, Randomized, Clinical Trial of GnP Combined with SBRT and Serplulimab Versus GnP as First-Line Treatment for Patients with Recurrent or Metastatic Pancreatic Cancer (WGOG-PAN 006/ICSBR-2).

[171] National Library of Medicine (2025). A Study Comparing BMS-986504 in Combination with Nab-Paclitaxel and Gemcitabine Versus Placebo in Combination with Nab-Paclitaxel and Gemcitabine in Participants with Untreated Metastatic Pancreatic Ductal Adenocarcinoma with Homozygous MTAP Deletion (MountainTAP-30).

[172] National Library of Medicine (2021). Safety and Efficacy of Immuncell-LC with Gemcitabine in Resectable Pancreatic Cancer.

[173] National Library of Medicine (2023). A Study of OT-101 with mFOLFIRINOX in Patients with Advanced and Unresectable or Metastatic Pancreatic Cancer (STOP-PC).

[174] National Library of Medicine (2026). Study of Daraxonrasib and Daraxonrasib + GnP as First-Line Treatment in Patients with Metastatic Pancreatic Adenocarcinoma (RASolute 303).

[175] National Library of Medicine (2025). Clinical Trial Comparing TQB2868 Injection Combined with Anlotinib Hydrochloride Capsules with Placebo Combined with Chemotherapy as First-Line Treatment for Metastatic Pancreatic Ductal Adenocarcinoma (mPDAC).

[176] National Library of Medicine (2025). Study of Daraxonrasib (RMC-6236) in Patients with Resected Pancreatic Ductal Adenocarcinoma (PDAC) (RASolute 304).

[177] National Library of Medicine (2022). Study of Nab-Paclitaxel and Gemcitabine with or Without SBP-101 in Pancreatic Cancer (ASPIRE).

[178] National Library of Medicine (2025). Study of NABPLAGEM vs. Nab-Paclitaxel/Gemcitabine in BRCA1/2 or PALB2 Pancreatic Cancer (PLATINUM-CAN).

[179] National Library of Medicine (2026). A Study to Evaluate Chemotherapy with or Without INCB161734 in Previously Untreated, KRAS G12D-Mutated Metastatic Pancreatic Ductal Adenocarcinoma (DAWN-303).

[180] National Library of Medicine (2025). Testing Higher Dose Radiation Therapy for Locally Advanced Pancreatic Cancer (LAP100).

